# Genome and transcriptome analysis of rock-dissolving *Pseudomonas* sp. NLX-4 strain

**DOI:** 10.1186/s40643-022-00548-w

**Published:** 2022-06-01

**Authors:** Yanwen Wu, Ayyappa Kumar Sista Kameshwar, Bo Zhang, Feifei Chen, Wensheng Qin, Miaojing Meng, Jinchi Zhang

**Affiliations:** 1 Co-Innovation Center for Sustainable Forestry in Southern China, Jiangsu Province Key Laboratory of Soil and Water Conservation and Ecological Restoration, Nanjing Forestry University, 159 Longpan Road, Nanjing, 210037 Jiangsu China; 2 Department of Biology, Lakehead University, 955 Oliver Road, Thunder Bay, ON P7B 5E1 Canada; 3 Department of Biology, University of Miami, Coral Gables, FL 33124 USA; 4 Present Address: Learning Support Team, St Margaret’s School, Victoria, BC V8X 3P7 Canada

**Keywords:** Rock mining, Ecological restoration, Silicate rock-dissolution, *Pseudomonas* sp. NLX-4 strain, Genome sequencing, Transcriptome sequencing

## Abstract

Microbial weathering processes can significantly promote soil properties and reduce rock-to-soil ratio. Some soil-inhabiting bacteria exhibit efficient rock-dissolution abilities by releasing organic acids and other chemical elements from the silicate rocks. However, our understanding of the molecular mechanisms involved during bacterial rock-dissolution is still limited. In this study, we performed silicate rock-dissolution experiments on a *Pseudomonas* sp. NLX-4 strain isolated from an over-exploited mining site. The results revealed that *Pseudomonas* sp. NLX-4 strain efficiently accelerates the dissolution of silicate rocks by secreting amino acids, exopolysaccharides, and organic acids. Through employing genome and transcriptome sequencing (RNA-seq), we identified the major regulatory genes. Specifically, 15 differentially expressed genes (DEGs) encoding for siderophore transport, EPS and amino acids synthesis, organic acids metabolism, and bacterial resistance to adverse environmental conditions were highly up-regulated in silicate rock cultures of NLX-4 strain. Our study reports a potential bacterial based approach for improving the ecological restoration of over-exploited rock mining sites.

**Graphical Abstract:**

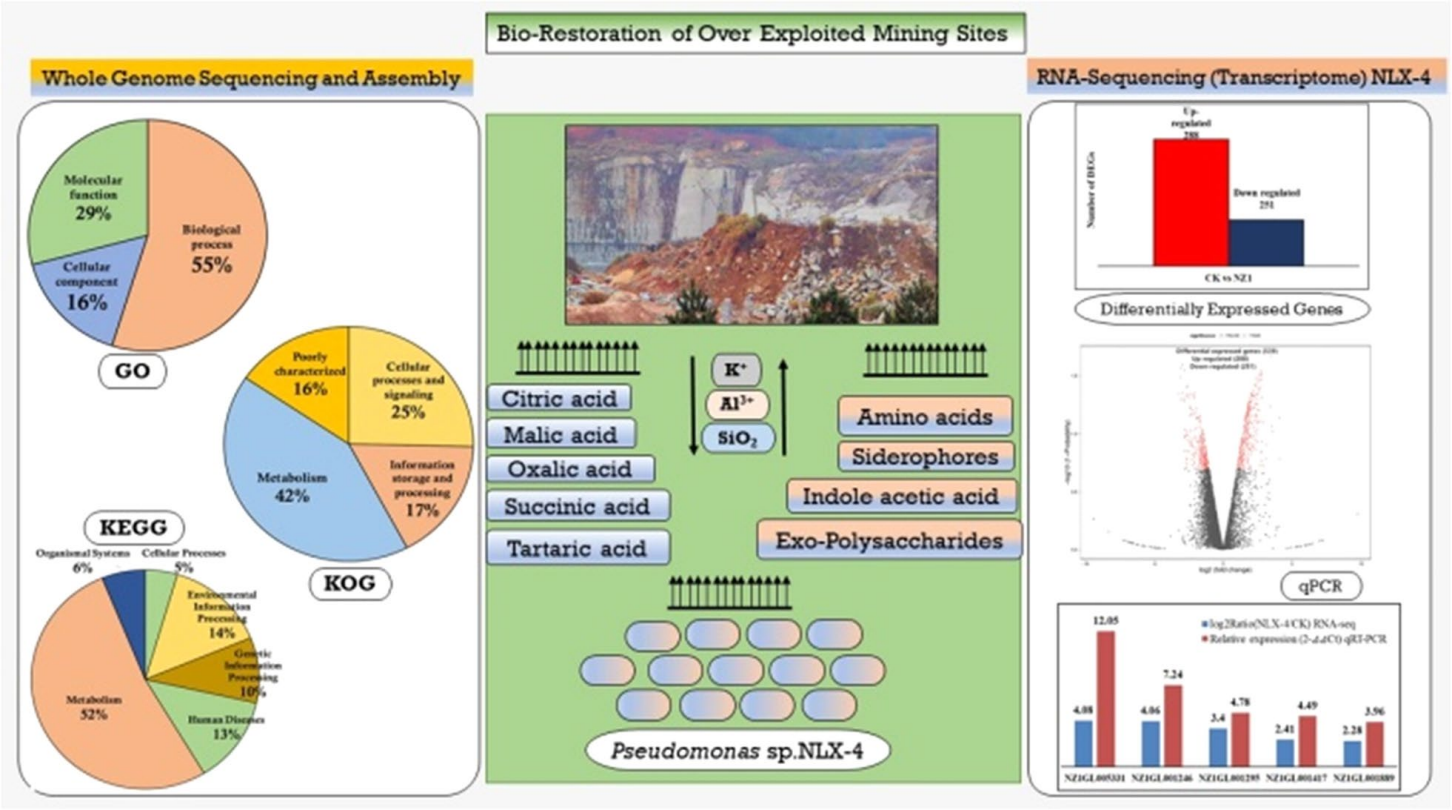

**Supplementary Information:**

The online version contains supplementary material available at 10.1186/s40643-022-00548-w.

## Introduction

About 90% of the earth crust is composed of silicate minerals and these silicates are building blocks of most rock types (Wang et al. 2021). Human encroachments of earth surface in the form of mining have severely destroyed the ecology of the mining areas, causing soil erosion, exposure of bedrock and loss of productivity (Comino et al. 2016; Erkossa et al. 2015; Ochoa‐Cueva et al. 2015). The area of soil and water loss in mining sites caused by overexploitation is increasing at a rate of 1.5 × 10^4^ km^2^ year^−1^, and more than 20% of these areas have been classified as rocky desertification (Balland-Bolou-Bi and Poszwa 2012). Studies were being conducted to resolve the environmental destruction of these mining sites by developing efficient ecological restoration technologies (Sun et al. 2018). Till date a variety of restoration methods were developed and implemented based on resource availability and ecosystem adaptability. Highly used restoration methods include a combination of these treatments: (a) in situ improvement of the physical and chemical substrate conditions; (b) spreading of the healthier soil and top-soiling methods; (c) spraying with herbaceous plant species and stabilization of soil; and (d) planting of trees and shrubs (Andrés and Mateos 2006). Spraying material on the surface of exposed rocks supplemented with plant seeds, soil and nutrients, which is known as external-soil spray seeding technology, is one of the most efficient and highly implemented approaches in the ecological restoration of mining areas (Deng et al. 2022). However, the current spraying material cannot break down rocks to continuously provide essential nutrients for plant growth. Therefore, soil formation for sustainable growth of plant is the key to solve the problem in long-term maintenance of external-soil spray seeding technology.

Microorganisms are widely present in natural environments. They break down the organic and inorganic compounds, and release them back into the environment as metabolic compounds (Gentry et al. 2021). This process is known as bioweathering that promotes the rock-to-soil transition and improves the soil environment for plant growth (Gleeson et al. 2006; Vandevivere et al. 1994). Studies have reported that microorganisms accelerate the process of bioweathering through chemical dissolution, formation of rock-biofilm and chelating reactions (Finlay et al. 2009; Lian et al. 2008). Various microbial proteins have been proven that play vital roles in the bioweathering of rocks. For example, the bacterial membrane transport/channel proteins are crucial in increasing the absorption and transportation rate of mineral elements during the process of rock dissolution (Chen et al. 2008). Conversely, the unavailability of minerals and nutrients in rocky areas forces the microorganisms to regulate the expression of corresponding genes that control the synthesis of proteins involved in rock solubilization (Alahari and Apte 2004).

Comprehensive understanding of the microbial gene regulation is of great concern in developing efficient microorganisms with rock-dissolving abilities (Finlay et al. 2009). Xiao et al (2012a) have conducted a PCR-based suppression-subtractive hybridization (SSH) experiments for exploring the differential expression of cDNA fragments in the control and experimental transcriptomes of *Aspergillus fumigatus*, cultured on potassium supplemented rock-dissolution growth medium (Xiao et al. 2012c). Xiao et al. (2012b) have also conducted a two-dimensional gel electrophoresis (2DGE) to understand the expression patterns of *Bacillus mucilaginosus* and its extracellular proteins secreted during the process of potassium rock dissolution (Xiao et al. 2012a). Nevertheless, it is difficult to draw convincing conclusions by analyzing the effect of individual factors on few candidate genes. Therefore, genome-wide transcriptome sequencing (RNA-seq) should be employed to capture the cellular snapshot of a rock-dissolving microorganisms at a given conditions (Kawahara et al. 2012).

Next-generation sequencing technologies have been applied for analyzing and revealing the complete gene expression patterns of an organism for understanding the molecular mechanisms underlying its biological process (Kawahara et al. 2012; Wang et al. 2009). Wang et al (2015) have conducted a high-throughput RNA sequencing study to understand the molecular mechanisms of potassium feldspar-dissolving *Aspergillus niger* (Wang et al. 2015). The *A. niger* genes encoding for proteins involved in synthesis and transportation of organic acids, polysaccharides, cystathionine beta-synthase, cysteine synthase, and glutathione synthase were found to be highly up-regulated (Wang et al. 2015). Recently, quantitative proteomics technology was combined with electrochemical analysis and vapor diffusion crystallization experiments to reveal how oxidoreductases produced by a *Bacillus subtilis* accelerate the weathering of serpentine at a protein-level (Liu et al. 2021). However, studies related to microbial weathering of silicate minerals were not fully researched till date. Our present research is focused on understanding and exploring the effects and participating genes of an efficient silicate rock-dissolving bacterial strain. We have isolated 22 bacterial strains from a disturbed silicate rock mining site. The most efficient rock-dissolving bacterium was screened out to understand the molecular complexities of silicate rock solubilization. We have conducted a whole-genome sequencing followed by genome-wide transcriptome analysis and validated the results using real-time fluorescent quantitative PCR (RT-qPCR) (Jiang et al. 2011). We propose that the experimental and sequencing data generated and analyzed in this study provide the efficient bacterial and genetic resources to improve the external-soil spray seeding technology for silicate rock mining areas.

## Materials and methods

### Rock samples

The silicate rock samples used in our study were obtained from Mount Lu (29°26′-29°41′N, 115°52′-116°08′E) in Jiangxi Province, southeast region in China (Additional file 1: Fig. S1). This area was a typical over-exploited silicate rock mining site with severely damaged ecological environment. The rock samples were fully rinsed in distilled water, dried and ground for 200 mesh sieve (Wu et al. 2017c). We have used X-ray diffractometer (XRD, ARL EQUINOX 1000, Thermo Fisher Scientific, USA) to analyze the composition of the rock samples. We have also conducted a rock mineral sheer analysis of the rock samples.

### Screening of microbial strains

Bacterial strains were isolated from the soil around the weathered rocks in Mount Lu using continuous gradient dilution method (Wu et al. 2017b). The isolated bacteria were cultured on Alexander Rove agar plates (5.0 g sucrose, 2.0 g Na_2_HPO_4_, 0.5 g MgSO_4_·7H_2_O, 0.005 g FeCl_3_, 0.l g CaCO_3_, l.0 g rock sample, 15.0 g agar, 1000 mL deionized water). Approximately, 200μL of each dilution (10^–4^, 10^–5^, 10^–6^) was spread onto the surface of Alexander Rove agar plate. The inoculated plates were incubated at 28 ℃ for 24–72 h to isolate individual colonies based on their size, color and morphology. Selected colonies were further purified using standard re-culturing techniques.

Each bacterial isolate was then tested for their capacity to produce indoleacetic acid (IAA) and siderophore, using the standard Salkowski colorimetric method and CAS assay, respectively (Bric et al. 1991). The CAS assay was monitored using a spectrophotometer set at an absorbance of 630 nm, the treatment group (with bacteria) and control group (without bacteria) were represented as A and Ar, respectively. The siderophore production was calculated as A/Ar ratio (represented as ±), the A/Ar ratio is inversely proportional to the siderophore production thus, lower A/Ar ratio represents more siderophore production (Wu et al. 2019). Strains with more than 10 mg L^−1^ IAA and +  +  + siderophore productions were chosen for further experiments.

The above selected bacterial isolates were cultured in 10 mL Luria–Bertani broth (LB) medium incubated for 24 h at 180 rpm in a shaking incubator. After 24 h, 1.0 mL of each isolate was inoculated into a conical flask containing 20 mL of fermentation medium (10 g saccharose, 2.0 g Na_2_HPO_4_, 0.5 g MgSO_4_·7H_2_O, 0.5 g (NH_4_)_2_SO_4_, 0.1 g NaCl, 0.1 g CaCO_3_, and 1000 mL deionized water) supplemented with 0.2 g silicate rock sample. The inoculated samples were incubated at 30℃ with 180 rpm for 7 days in a shaking incubator, conical flasks with 1.0 mL of inactivated isolates are considered as control (Wu et al. 2017c). The amount of SiO_2_ released from rock sample was determined using the standard silico-molybdenum blue spectrophotometric method (Meyer and Bloom 1993). The NLX-4 strain which exhibited excellent IAA, siderophore and SiO_2_ releasing abilities was selected for genomic analysis.

### Identification of strain NLX-4

Genomic DNA of NLX-4 strain was successfully extracted using CTAB method (Wu et al. 2017a). The 16S rRNA gene was PCR amplified using the following forward 27F (5′-AGAGTTTGATCC/ATGGCTCAG-3′) and reverse primers1492R (5′-TACGGTTACCTTGTTA CGACTT-3′). The PCR products were verified using 1% agarose gel, and further sequenced by Genscript Co. LTD (Nanjing, China). The 16S rRNA gene sequence of NLX-4 strain was submitted to the NCBI (http://blast.ncbi.nlm.nih.gov/) database for identifying the phylogenetic closeness of the NLX-4 strain with others using BLAST. The phylogenetic relationship of NLX-4 strain was analyzed using MEGA 7.0 software, using neighbor-joining phylogenetic tree method with Bootstrap values greater than 50% and 1000 replications were used for generating the phylogenetic tree (Sohpal et al. 2010).

### Rock-dissolution experiments

The culture medium supplemented with 5.0 g L^−1^ silicate rock sample (sole K^+^ source) was inoculated with NLX-4 strain and incubated at 30 ℃ with 180 rpm in a shaking incubator for different time periods ranging from 0, 2, 5, 9, 12, 15, 22, and 30 days, respectively. The culture medium was regularly monitored (the above-mentioned time periods) for determining the concentration of K^+^ and Al^3+^ ions, using inductively coupled plasma-atomic emission spectrometry (ICP-AES, Vista MPX, Varian, USA) as per the protocol reported by Wu et al. (2017c) and Kanicky and Mermet (1997). The concentration of Si ion released from the rock cultures was measured using the silico-molybdenum blue spectrophotometric method as mentioned above. The exopolysaccharides (EPS) secreted by NLX-4 strain was determined using the standard ethanol precipitation method (Sambrook and Russell, 2006). Amino acids and organic acids were analyzed using high-performance liquid chromatography (HPLC, 1260 Infinity, Agilent Technologies, USA) using the well-established protocols (Wu et al. 2017b). The rock culture samples inoculated with inactivated NLX-4 strain was considered as control. We have also determined the particle diameter of the rock samples by retrieving the bacteria–rock mixtures and the rocks were separated based on the median particle diameter (D_50_) and analyzed using laser particle size analyser (LDSA, S3500 SI, Microtrac, USA) (Wu et al. 2017c). The particle diameter variations (PDV) were calculated as the differences between the experimental group and the control group.

### Whole-genome sequencing and assembly of NLX-4

The genomic DNA of NLX-4 strain was extracted using the standard SDS-based method (Zhou et al. 1996) and sequenced using the Illumina Hiseq 4000 (Illumina, Inc., San Diego, CA) and PacBio RSII sequencing platform (BGI, Beijing, China). The raw data produced by Illumina Hiseq 4000 were initially processed to obtain clean data by discarding the low-quality reads (reads containing more than 36 bases, reads with ≤ 20 base-qualities, reads with ploy-N ≥ 9 bp, reads with adapter or duplication bases). Similarly, the raw data of NLX-4 genome obtained from PacBio RSII were filtered by removing the polymerase reads shorter than 100 bp or the read mass less than 0.80, then the subreads with length ≥ 1000 bp and reads without adapters were extracted from filtered polymerase reads, respectively. The clean reads obtained after filtering were assembled using RS_HGAP Assembly3 (SMRT Analysis v2.3.0). The correct K-mers were observed for the reads with deep sequencing (high frequency), while random sequencing error-containing K-mers were observed for reads with low read frequency (Li et al. 2010). The error correction method used in this study was based on K-mer frequency information. The K-mer size was determined by the genome size, read length, and supercomputer memory (Li et al. 2010), thus K = 15 bp was chosen here. The corrections of single-base error in contig were performed using two-round analysis methods to process the data from Illumina Hiseq 4000. The first-round error correction method was carried out by soap SNP and soap Indel software, while the second-round of error correction was conducted by GATK analysis pipeline. The circle form by contigs was verified by checking the overlap using SSPACE-LongRead to determine the subreads or corrected reads based on the data obtained from PacBio RSII (Boetzer and Pirovano 2014). The assembly of the bacterial genome was inspected by: (1) comparing 100× clean data to the assembly result (reads utilization > 95%, the depth of backward and forward bunches > 3×, the insert size of backward and forward bunches was distributed within the proper range, satisfied randomness, and the depth of single bunches = 0); (2) carrying out the statistics of repetitive sequence (parameters were set as -e 1e−10); (3) performing GC-depth assessment. Gene annotation was mainly based on amino acid sequence alignment (Wyman et al. 2004). The amino acid sequence of the gene was compared with the BLAST database, and the corresponding functional annotation information was obtained. Since each of these sequence comparisons had more than one result, the optimal comparison was retained as the annotation of gene, so as to ensure its biological significance (Slater and Birney 2005). All annotations were completed by using BLAST software to combine various databases, including Gene Ontology (GO), Clusters of Orthologous Group (COG), Kyoto Encyclopedia of Genes and Genomes (KEGG), Swiss-Prot, and NCBI non-redundant protein (nr).

### RNA extraction, library preparation and RNA-seq

The NLX-4 strain samples cultured with rock sample (5.0 g L^−1^) growth medium were incubated at 30 ℃ with 180 rpm for 15 days, while strain NLX-4 cultured in fermentation medium added with 0.3 g L^−1^ KCl was set as control. To further investigate the molecular mechanisms resulting involved in rock-dissolution we have performed a genome-wide RNA sequencing. The total RNA extraction was performed using TRIzol® Invitrogen reagent (Invitrogen, Carlsbad, CA). The total RNA extraction of NLX-4 strain cultures was performed separately for the control and treated group cultures (Gómez‐Lozano et al. 2012). RNA quality was analyzed using Agilent® bioanalyzer (Agilent 2100, Agilent Technologies, USA). Ribo-Zero rRNA removal kit (Illumina, Inc. San Diego, CA) was used for extracting total RNA devoid of rRNA. After extracting sequencing quality total RNA we have added fragmentation buffer was for breaking down the mRNA to short fragments. Taking these short fragments as templates, random hexamer-primers were used to synthesize the first-strand cDNA. The second-strand cDNA was synthesized using buffer supplemented with dATPs, dGTPs, dCTPs, dUTPs, RNase H and DNA polymerase I, respectively, after removing dNTPs. Short fragments were purified using the QiaQuick® PCR extraction kit (QIAGEN, Hilden, Germany) and later resolved using the elution buffer for repairing the ends of the strands and adding poly (A) tails. After that, the short fragments were connected with sequencing adapters. Then, the UNG enzyme was used to degrade the second-strand cDNA, followed by purifying the product using the MinElute® PCR Purification Kit (QIAGEN, Hilden, Germany) before the PCR amplification process. Thus, obtained mRNA library was sequenced using Illumina HiSeq2000 (Illumina, Inc., San Diego, CA).

### Bioinformatic analysis of NLX-4 transcriptome data

Raw data produced by Illumina Hiseq 2000 were initially processed to obtain clean data by discarding the low-quality reads (reads containing adapter sequences, ploy-N, or low-quality bases). Clean reads obtained after filtering were mapped to the NLX-4 reference genome (fully assembled genome of strain NLX-4) using SOAP2 (Li et al. 2009). Mismatches with no more than 5 bases were allowed during the process of alignment. Reads per kb per million reads (RPKM) method was used to calculate individual gene expression of the test and control samples (Mortazavi et al. 2008). The RPKM level data of control and treatment samples were also analyzed using edgeR, limma, and Glimma statistical analysis packages to obtain statistically significant DEGs (Audic and Claverie 1997). DEGs with false discovery rate (FDR) ≤ 0.001 and fold change ratio larger than 2 were chosen for gene ontology (GO) functional enrichment analysis and KEGG pathway analysis. Only the genes exhibiting the *P*-value < 0.05 and Q-value < 0.05 were considered as differentially expressed (Tribelli et al. 2015).

### Quantitative real-time PCR (RT-qPCR) validation

We have specifically performed the RT-qPCR analysis using the significant list of differentially expressed RNA transcript sequences encoding for 5 metabolically important genes. The forward and reverse primers were designed using the Primer Premier 5.0 software (Additional file 1: Table S6a). RT-qPCR procedures were performed as previously suggested (Tribelli et al. 2015). The relative quantification of the control and test samples were performed by including three biological replications and the obtained gene expression results were normalized to the reference gene (16S rRNA). The differential expression of the subjected genes was calculated according to the standard 2^−ΔΔCt^ method (Livak and Schmittgen 2001).

### Statistical analysis

All experiments were conducted in triplicates to obtain statistically significant results. One-way analysis of variance (ANOVA) and two independent samples *t*-test were carried out to quantify the significant differences between different conditions. Pearson correlation analysis was performed to assess the effects of different factors on rock dissolution and release of elements. Statistical analysis was accomplished using the IBM SPSS 22.0 and R 3.3.0 software.

## Results and discussion

### Screening of the efficient strain

To confirm the type of rock used for the efficient strain screening, we analyzed the composition of the selected rock samples. The results revealed the percentages of potassium feldspar, quartz, and mica in isolated rock samples were 52%, 36%, and 10% by mass, respectively. The rock mineral sheers analysis showed that SiO_2_, Al_2_O_3_, and K_2_O accounted for 73.8%, 13.4%, and 9.9% by mass, respectively, which proved that the selected rock samples are silicate rocks.

**Fig. 1:**
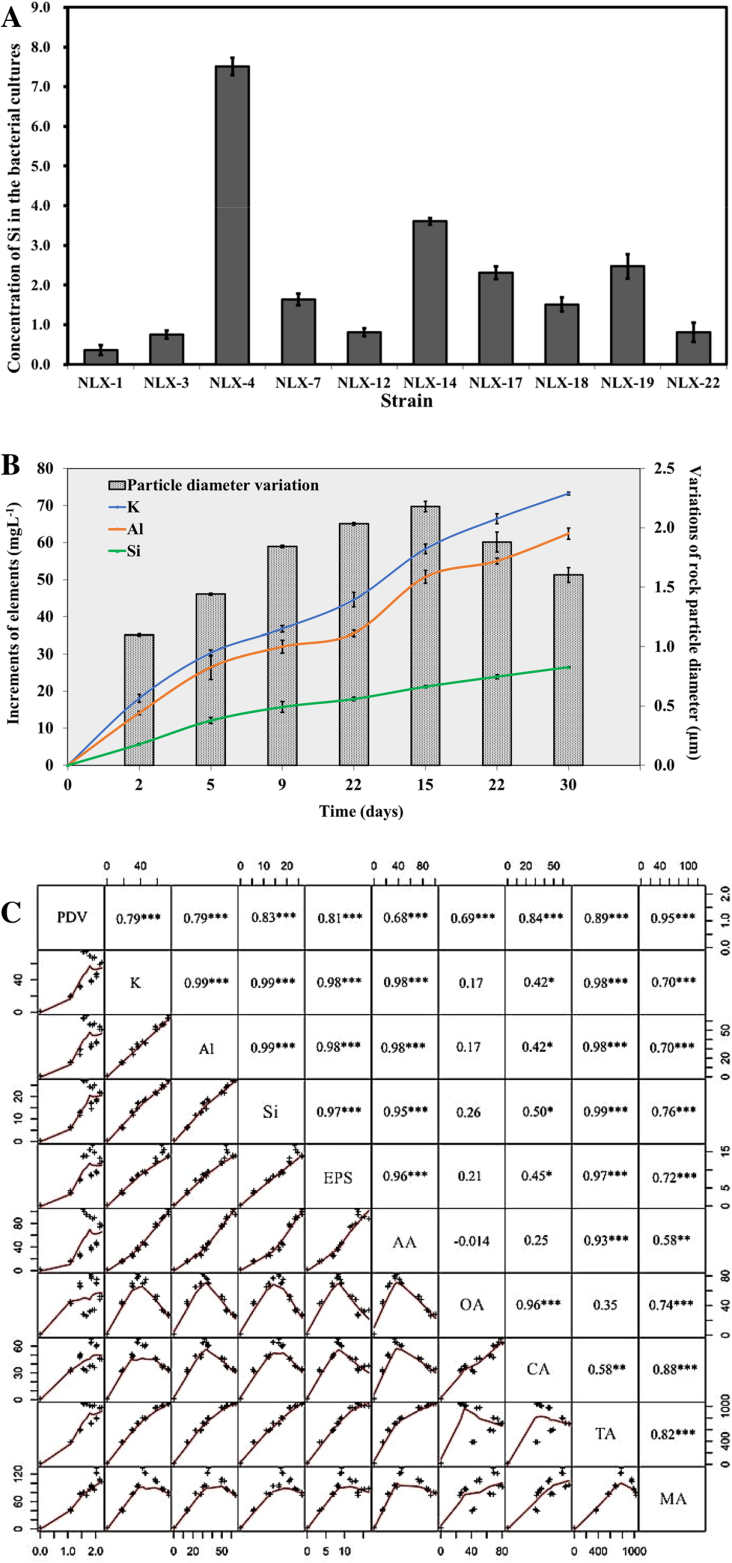
**a** The fermentation results of different bacterial strains (10 strains in total) for 7 days showing the final concentration of Si released during the fermentation of rock samples; **b** the final concentrations of released elements K^+^, Al^3+^ and Si^4+^ from the rock samples by the NLX-4 strain and particle diameter variations (PDV); **c** Pearson’s correlation analysis of PDV of the fermented rock samples; exopolysaccharides (EPS); acetic acid (AA); oxalic acid (OA); citric acid (CA); tartaric acid (TA); malic acid (MA). Symbols *, **, and *** represent that the correlations are significant at 0.5, 0.01, and 0.001 levels, respectively. Error bars represent the standard deviation (*n* = 3)

Studies have proved the interaction between the IAA producing bacteria and plants has various applications to the plants ranging from pathogenesis to the phyto-stimulation (Spaepen et al. 2007). The siderophore secreted by bacteria has been proved to have a variety of vital capacities (Crosa and Walsh 2002). For example, it can inhibit the reproduction of pathogenic microorganisms causing plant diseases (Sahu and Sindhu 2011), chelate metal ions (Neilands 1995), and promote mineral decomposition (Neubauer et al. 2002). Therefore, the rock-dissolving abilities of 22 different microbial isolates were considered for further studies which were evaluated for their ability to produce IAA and siderophore (Additional file 1: Table S1). The highly efficient strains producing IAA more than 10 mg L^−1^ (Arancon et al. 2004) and exhibiting strong siderophore productivity (+ + +) (A/Ar: 0–0.6) were considered for further studies (Hesse et al. 2017). Based on the IAA and siderophore producing abilities, ten bacterial strains NLX-1, 3, 4, 7, 12, 14, 17, 18, 19 and 22 were further considered for the rock-dissolution experiments. The rock-dissolving capacity of a microbial strain is directly proportional to the concentration of silicon released from the rocks (in the form of SiO_2_ in the fermentation broth). The rock-dissolving ability was calculated by finding the difference between the experimental and control groups, respectively (Fig. 1a). The NLX-4 strain exhibited the significant advantageous effect in the release of Si (*P* < 0.001). It was reported that the *Pseudomonas* sp. strains were among these typical bacteria which produce both the IAA and siderophores (Bano and Musarrat 2003; Gupta et al. 2002; Rajkumar et al. 2005). It also showed that the release of Si from rock by NLX-4 increased by 7.51 mg L^−1^ compared to control after 7 days of cultivation, which was remarkably higher than the amount of Si-releasing induced by reported *Pseudomonas* strains (Maurice et al. 2001). Thus, we have selected NLX-4 strain for further genomic studies.

### Identification of silicate rock-dissolving strain NLX-4

The standard morphological experiments of NLX-4 strain showed NLX-4 strain is a rod-shaped Gram-negative bacterium with single flagella (Additional file 1: Fig. S2a). To further identify the NLX-4 strain, sequenced 16S rRNA gene of NLX-4 strain was submitted to NCBI GenBank with an accession number KX379232 (Additional file 1: Fig. S2b). Results obtained from BLAST and the phylogenetic tree analysis endorsed that NLX-4 strain belonged to the *Pseudomonas* genus (Additional file 1: Fig. S2). Based on the results of the morphological and phylogenetic analysis, NLX-4 strain was identified as a *Pseudomonas* sp. strain.

### Effects of Pseudomonas sp. NLX-4 on rock-dissolution

The final concentrations of K, Al, and Si released from silicate rock samples by NLX-4 strain showed an increasing trend, while the particle diameter variation of rock samples increased significantly in 0 to 15 days cultures followed by a slight decline in the PDV values in the later time periods, respectively (Fig. 1b). We have observed a gradual increase in release of elements from 2^nd^ to 30^th^ day cultures of NLX-4 strain, respectively (Fig. 1b). The concentrations of K, Al and Si (mg L^−1^) elements released by NLX-4 reached a maximum by the 30th day with 73.27 mg L^−1^, 62.43 mg L^−1^, and 26.50 mg L^−1^, respectively. The highest value of PDV was observed on 15th day cultures of NLX-4 (2.18 mm), which later reduced to 1.60 mm by 30th day, respectively (Fig. 1b).

To explore the effective components of NLX-4 strain promoting the rock dissolution, we have analyzed and compared the final concentrations of exopolysaccharides, amino acids, and organic acids secreted in fermentation broth (Additional file 1: Table S2). Pearson analysis was used to assess the correlation between PDV, element concentrations, EPS, amino acids, and organic acids, respectively (Fig. 1c). Apparently, PDV of rock samples has highly significant correlations (*P* < 0.001) with the release of K, Al, Si (Fig. 1c). Meanwhile, EPS, amino acids, and all organic acids exhibited highly significant correlations (*P* < 0.001) with PDV, with malic acid being the most relevant factor (Fig. 1c). In addition, EPS, amino acids, tartaric acid, and malic acid showed highly significant correlations (*P* < 0.001) with the release of three elements (Fig. 1c). In the study conducted by Welch and Vandevivere (1994), EPS did not affect mineral dissolution when it worked alone. However, EPS freshly secreted by bacteria appeared the capacity to accelerate the process of mineral dissolution by forming metal–organic complexes at mineral surface to weak the metal–oxygen bond in its structure (Tourney and Ngwenya 2014). During the process of rock dissolution, the EPS and amino acids produced by bacterial strain were proven to have the capacity to destroy the crystal structure of rocks, which attains strength in the acidic environments (Braissant et al. 2003). The organic acids secreted by a bacterial strain not only create favorable acidic environmental conditions for the dissolution of the rock, but also have advantages over inorganic acids to dissolve rock due to their complexation with the cations in the mineral crystal lattice of rocks (Wu et al. 2017b; c). In this study, the oxalic acid might show more complexation than acid solubilizing capacity to release K, Al, and Si, while the other three acids had different degrees of complexation with these elements. Effective and persistent microbial mineral bioweathering is dependent on bacterial acid production and tolerance of bacteria. A recent study conducted on *Pseudomonas azotoformans* F77 has revealed the silicate mineral weathering and acid tolerance capacities by culturing it on biotite (silicate mineral) (Li et al. 2021). Yuan-Li et al. also has revealed the genome, transcriptome and genetics of *P. azotoformans* F77 strain, explained the molecular mechanisms underlying the silicate mineral bioweathering and acid tolerance by deleting the genes involved in gluconic acid metabolism and acid tolerance mechanisms (Li et al. 2021). This study has reported that genes involved in acid production and tolerance were highly expressed when cultured on biotite.

### Sequencing and assembly of NLX-4 strain

The genome of NLX-4 strain was sequenced and de novo assembled by implementing a hybrid approach involving PacBio RSII and Illumina HiSeq 4000 sequencing systems. The quality control analysis on the raw read sequences has resulted in 713,706, 632 base pairs of clean sequence data and total of 79,379 sequences with a mean read length of 8991 bp (Additional file 1: Table S4). Thus, obtained filtered subreads were further subjected to de novo assembly using RS_HGAP Assembly v3 in SMRT® analyser v2.3.0 software. The assembly of the PacBio sequence data was performed using the soapSNP and soapIndel by using the Illumina HiSeq 4000 sequence data.

The sheared genomic DNA of NLX-4 was also subjected to Illumina HiSeq 4000 sequencing system by constructing a 300-bp insert library with 2 × 100 bp read length. The Illumina sequencing has resulted in 904 Mb of raw data with an adapter and duplication percentages to be 0.23 and 8.03, respectively, and total reads were 9,049,948 (Additional file 1: Table S4 and S6b). The quality control analysis has resulted in clean data of 817 Mb of sequence data with filtered reads and low-quality filtered reads percentages were 9.68 and 1.39, respectively (Additional file 1: Table S4 and S6b). The final NLX-4 genome sequence assembly resulted in single chromosome with 0 gaps, single base quality of 1, structure base and reads usage percentages were 0.9975 and 0.9915, respectively, at a genome depth of 40.75 and genome size of 6.52 Mb (Additional file 1: Table S5a). The gene prediction of the NLX-4 genome sequence was performed using the Glimmer v3.0.2 and the whole-genome sequence was analyzed for the annotations using BLAST with non-redundant protein database (NR), Swiss-Prot, TrEMBL, COG, KEGG, InterPro and GO databases. The genome was also analyzed using the following servers RNAmmer, tRNAscan, Rfam, IslandPath-DIOM, SIGI-HMM, IslandPickerp and CRISPR Finder servers, respectively (Additional file 1: Fig. S3–S5).

The genome of *Pseudomonas* NLX-4 strain is a single circular chromosome with 6.7 Mb (6,771,445 bp) length and 63.21% of G + C content (Additional file 1: Table S6b). The genome contains 6,239 protein coding genes with an average length; internal length and internal GC content were 949 bp, 852,628 bp and 56.55%, respectively (Additional file 1: Table S5b). It also contains 72 tRNA genes, 19 rRNA genes and 17 sRNA genes. The NLX-4 genome has also showed sequencers for an intact prophage sequence with a length of 39,233 bp (start: 5,375,574, end: 5,414,806) with a GC% of 61.36. The results from Tandem Repeat Finder software shows that the genome contains a total of 192 tandem repeat fragments (TRF), 90 minisatellite and 36 microsatellite DNA sequences with total lengths of 35,083 bp, 4,597 bp and 1380 bp, respectively (Additional file 1: Table S5d). Total of four CRISPR (ID-1, 2, 3 and 4) spacer sequences with a sequence length ranging between 34, 35 40 and 48, respectively, and gene Island analysis has resulted in 54 gene islands (Additional file 1: Table S5e).

**Fig. 2:**
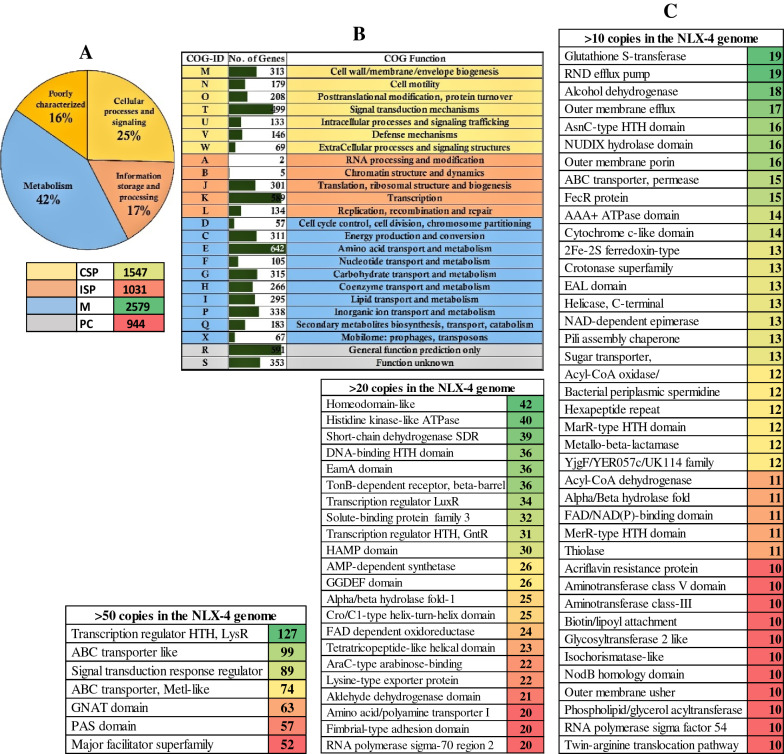
**a** Genome-wide distribution of Prokaryotic Orthologous Groups (COG) where CSP: cellular process and signaling 25% (1547); ISP: information storage and processing 17% (1031 genes); M: metabolism 42% (2579 genes); PC: poorly characterized 16% (944 genes). **b** The listed table illustrates the individual classification of genes in different COG classes which are differentiated based on the color as per pie diagram 2A. **c** Pictorial illustration of genes occurring > 50 copies, > 20 copies and > 10 copies in the *Pseudomonas* sp. NLX-4 strain genome

**Fig. 3:**
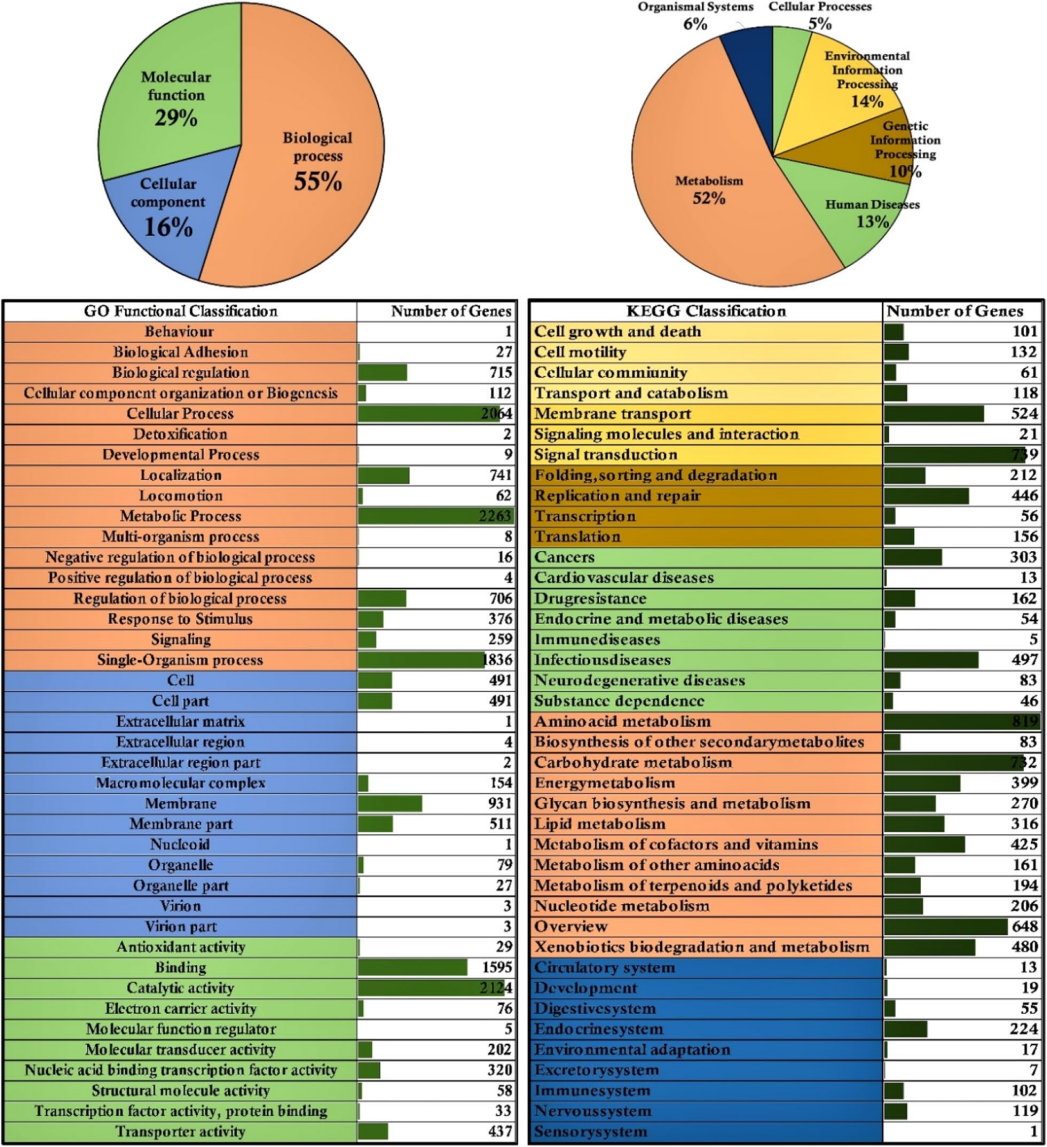
Pictorial illustration of genome-wide distribution of *Pseudomonas* sp. NLX-4 strain genes against gene ontology (GO) and KEGG database [Note: pie chart illustrates complete distribution of genes and tabular illustration lists the distribution of genes in individual classes. The color schema uses in pie chart is also applied for the tabular representation]

To benefit the further study on microbial weathering, a whole genomic sequencing was also conducted in our research. The whole genome was annotated using BLAST annotation pipeline by searching and retrieving the genome-wide annotations from different annotation databases such as prokaryotic orthologous groups (COG), gene ontology (GO), InterPro, KEGG, BLAST-NR, Swiss-Prot databases, respectively. The annotated genome of NLX-4 strain contains a total of 6041 (96.82%) protein coding gene sequences, with 5045 (80.86%) COG, 3996 (64.04%) GO, 5342 (85.62%) InterPro, 4386 (70.29%) KEGG, 6038 (96.77%) BLAST-NR and 3897 (62.46%) Swiss-Prot, respectively (Additional file 1: Table S5f). The COG has classified the protein encoding genes into 25%-cellular signaling and processing, 17%-information storage and processing, 42%-metabolism and 16%-poorly characterized, respectively (Fig. 2). The GO has classified protein coding genes into 55%-biological process, 16%-cellular process and 29%-molecular function, respectively (Fig. 3). The KEGG database has divided protein coding genes into 52%-metabolism, 6%-organismal systems, 5%-cellular processes, 14%-environmental information processing, 10%-genetic information and processing and 13%-human diseases, respectively (Fig. 3). Genes encoding for transcription regulator HTH (LysR), ABC transporter like, Signal transduction response regulator ABC transporter (Metl-like), GCN5-N-acetyltransferase (GNAT), PAS domain, major facilitator superfamily, homeodomain-like, histidine kinase, short-chain dehydrogenase, DNA-binding HTH, EamA domain, TonB-dependent receptor proteins and other genes were found to occur in multiple copies in the NLX-4 genome (Fig. 2).

### Rock-dissolution molecular mechanisms and genes

In NLX-4 genome, 2124 genes exhibit catalytic activity, 29 genes exhibit antioxidant activity, 1595 genes exhibit binding activity (Fig. 3). Genome of NLX-4 encodes genes involved in important metabolic pathways with 819-amino acid metabolism pathways, 83-biosynthesis of secondary metabolites, 732-carbohydrate metabolism, 399-energy metabolism, 270-glycan biosynthesis and metabolism, 161-metabolism of other amino acids, 194-metabolism of terpenoids and polyketides, 480-xenobiotic biodegradation and metabolism, respectively. The prokaryotic orthologous group classification of NLX-4 genome contains 311 genes in energy production and conversion, 642-amino acid transport and metabolism, 315-carbohydrate transport and metabolism, 183-secondary metabolite biosynthesis, transport and metabolism, respectively (Fig. 3). Metabolically, some of the important genes include siderophore encoding genes (NZ1GL004767; NZ1GL000887; NZ1GL005349), polysaccharide biosynthesis protein (NZ1GL001889), nitric oxide dioxygenase (NZ1GL001246) and other proteins which are involved in the biosynthesis of organic acids, amino acids and secondary metabolites required for the dissolution of rocky material.

### Transcriptome of NLX-4

The sequencing has resulted in a total of 143 million reads with each sample the number of reads varied between 23 to 24 million reads, respectively (Additional file 1: Table S7a and b). The quality control analysis was performed to discard the low-quality reads and obtain clean reads; these clean reads were further mapped both to the gene and genome of NLX-4 strain obtained above. The RNA sequence data of six samples (control and treatment (NLX-4) in triplicates) were aligned to the reference genome using SOAPv2.0. The sequence reads were aligned to the NLX-4 genome and non-ribosomal reads which did not align uniquely with the NLX-4 genomic sequences were removed. Total number of rRNA reads mapped to the NLX-4 reference genome varied from 0.10% to 1.51%, respectively. The alignment results obtained from gene and genome level are reported in Additional file 1: Table S7a and b, respectively. On average 90.53% of transcripts were found to be mapped to the genome of NLX-4, which demonstrates the suitability of using RNA sequencing data for further studies.

We have observed that in control samples the total number of rRNA reads count were slightly higher than the treatment samples, which might be due to the insignificantly higher rRNA to mRNA ratios in control samples. The present genome-wide transcriptome study of NLX-4 completely covered the majority of all the genes with more than one read. Higher reproducibility was observed among the biological replicates, they have similar number of total and the mapped reads (Additional file 1: Fig. S11–S13). The mapped reads were further subjected to a series of analysis including sequence assessment, biological contextualization, gene structure refinement, alternative splicing, novel transcript detection and SNP analysis (Additional file 1: Fig. S14). Transcriptome analysis conducted by edgeR, limma and Glimma packages showed that the strain NLX-4 cultured with/without silicate rocks resulted in 539 (288-up and 251-down) DEGs. Those DEGs were further analyzed for its biological contextualization using GO and KEGG pathway enrichment analysis (Additional file 1: Fig. S15).

### Differentially expressed genes

**Fig. 4:**
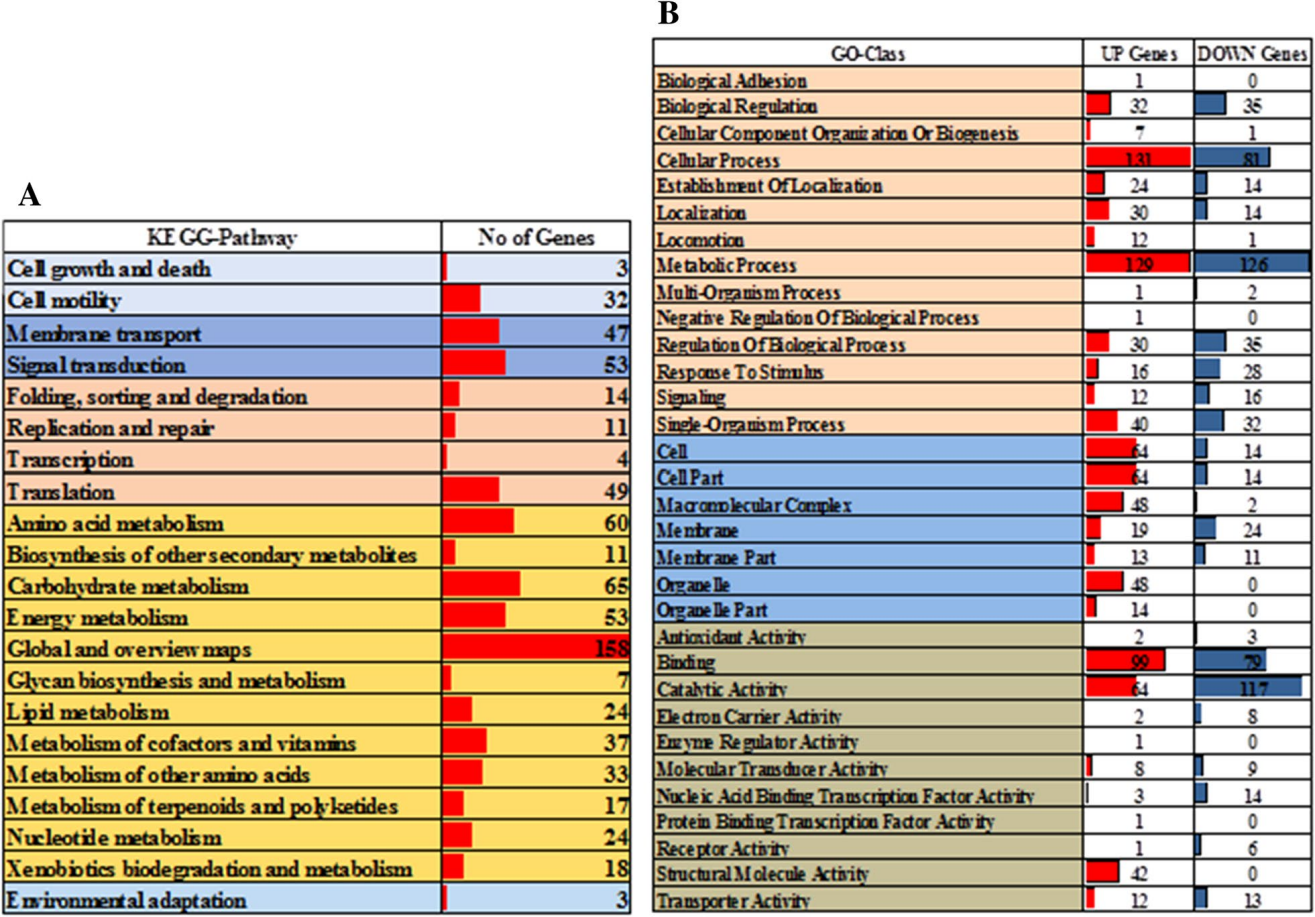
The total number of differentially expressed significant genes obtained from RNA-Seq with **a** enriched KEGG pathways and **b** Gene ontology (biological process, cellular component, and molecular function)

As the bacteria were previously reported to release nutrients from rocks for their growth (Alahari and Apte 2004), the rocks using as a kind of nutritional source were considered to have its influence on the metabolic process of bacteria (Xiao et al. 2012a). Compared to the samples composed of soluble K (KCl) as potassium source, the NLX-4 strain using K-bearing silicate rock as potassium source has resulted in higher EPS, amino acids, and organic acids. These differences illustrated that the interaction of NLX-4 strain and rock to release nutrients for bacterial growth significantly accelerated the process of rock dissolution (Wu et al. 2017c). The RPKM values obtained from the quantification of the control and treatment samples were used for finding the significant DEG. We have followed two different approaches for finding the DEGs among the samples (a) using the Audic Claverie method, (b) using a customized pipeline implementing edgeR, limma and Glimma packages. The results obtained from both the approaches have given us almost the same list of DEGs. Genes encoding for nitric oxide dioxygenase, flagellin, glutathione-S-transferase (GST), chaperonin GroES, heat shock protein HSP10, elongation factor prokaryotic, bacterioferritin, polysaccharide biosynthesis protein Epsc, DNA-methyl transferase, flagellar FliS, FlaG, FlgN protein, diguanylate cyclase, large ribosomal protein L7 and two-component system-NARXL were highly expressed with a fold change values log > 2.5 (Fig. 4 and Additional file 1: Fig. S15). While the genes encoding for fatty acyl-CoA synthase, sulfur dioxygenase, DNA polymerase III, acetyltransferase, cation symporter, sulfide: quinone oxidoreductase, alcohol dehydrogenase, pyrroloquinoline quinone biosynthesis protein B, hydrogen cyanide synthase (HcnB), CBS domain, cytochrome c peroxidase, polar amino acid transport system, cytochrome c oxidase and nitrate reductases were down-regulated in the treatment samples with a fold change value of log < 2.5, respectively (Fig. 4 and Additional file 1: Fig. S15). We have also analyzed the enrichment of the DEGs in biological pathways using KEGG pathways and GO analysis, respectively (Fig. 4 and Additional file 1: Fig. S15). RT-qPCR was used to further validate the expression level of genes identified in Illumina sequencing analysis. A total of 5 DEGs, encoding for basic amino acid, nitric oxide dioxygenase, GST, bacterioferritin, and polysaccharide biosynthesis protein, with relatively higher gene expressions were chosen as candidates for the RT-qPCR study (Additional file 1: Table S6a). These 5 DEGs showed a consistent pattern between Illumina sequencing results and RT-qPCR (Additional file 1: Fig. S17), which indicated that the transcriptome data are reliable and accurate.

As has been already known, a total of 15 genes involved in the regulations of ionophore transportation, EPS and amino acids synthesis, and organic acids metabolism were highly expressed with a fold change values log > 2.5. The bacterioferritin encoding gene (involved in ionophore and transportation of siderophore) (Carrondo 2003), was highly up-regulated in the tested conditions.

The gene ontology analysis of the DEGs showed that majority of these gene products are mainly involved in the process of protein translation, metabolic, and cellular macromolecule biosynthesis (Fig. 4). This study also proposes the involvement of EPS, amino acids and organic acid are associated with various factors playing crucial roles in protein translation, metabolic, and cellular macromolecule biosynthetic, such as elongation factors and large ribosomal protein L7 processes. In addition, the oxidoreductases such as nitric oxide dioxygenase were also found to regulate the amino acids and organic acid metabolism reactions (Litvinova et al. 2015). The up-regulation of genes involved in macromolecular assembly of extracellular proteins, polysaccharides, and glycoproteins, might be involved in creating a better weathering microenvironment during (a) bacterial attachment to rock particles; (b) formation of the bacterial–rock aggregates. Genes encoding for flagellin, polysaccharide biosynthesis protein Epsc, flagellar FliS, FlaG, FlgN protein, diguanylate cyclase (Chen and Schaap 2012) endorses the above functions. The gene encoding for DNA-methyl transferase with a function inducing the lipopolysaccharide, was found to be highly up-regulated (Zhang et al. 2013) and these results also confirms the existence of polysaccharides in the environment (Fig. 4). Recent study conducted by Picard et al. 2021, have reported about a novel pyrroloquinoline quinone (PQQ) cofactor independent glucose dehydrogenase enzyme involved in production of gluconic acid which is involved in mineral bioweathering of biotite (Picard et al. 2021). Picard et al. have conducted a genome analysis study on *Collimonas pratensis* strain PMB3 strain and developed three mutant strains which did not produce gluconic acid which has revealed that the mutant strains exhibited low mineral weathering abilities compared to wild type (Picard et al. 2021). Genes encoding for glucose dehydrogenase (GDH) and glucose/methanol/choline oxidoreductases were highly expressed in the NLX4-strain samples cultured on rock samples. Similarly, quinoprotein glucose dehydrogenase (gcd) genes were found to be differentially expressed among the datasets. Recent study conducted by Wang et al. (2020) on *Pseudomonas azotoformans* F77 strain and its bioweathering capacity of biotite (abundant mineral identified in acidic soil) (Wang et.al. 2020). This study has revealed the importance and involvement of gcd gene (involved in gluconic acid metabolism) and adh gene (involved in pilus formation) in biotite mineral weathering.

Glutathione (GSH) is essential for the cells to adapt to the deficiency of K^+^ deficiency by acting as an important antioxidant and detoxifying agent in cells (Wang et al. 2015). GST has the function to catalyze the conjugation of glutathione with a wide variety of electrophiles (Beckett and Hayes 1993). In the process of rock dissolving, various elements in the crystal lattice of rocks are released. Apart from cell biogenic elements, some heavy metal elements and impurities also enter into the bacterial cells as exogenous substances, which generate toxic effects such as inducing oxidative stress (Sun et al. 2013). Therefore, *Pseudomonas* sp. NLX-4 performs cell detoxification by up-regulating the expression of GST genes, and homeostasis in its cells can be maintained to overcome the harsh environment. GST has been found up-regulated in the bio-dissolving of K-bearing mineral affected by *Aspergillus fumigatus* (Xiao et al. 2012b), which is consistent with the results of this study. Furthermore, two-component system-NARXL in bacteria, which referred to numerous sensory-response circuits operate by making use of a phosphorylation control mechanism (Kobayashi et al. 2016), is also helpful for bacteria to be masters at adapting and coordinating cellular events to accommodate adverse conditions.

Moreover, as K^+^ is an essential element for the metabolism of bacteria because of its indispensability for synthesis of proteins and enzymes, *Pseudomonas* sp. NLX-4 may first cause incorrect folding of proteins and enzymes without K^+^ (Fresht et al. 1992). Therefore, amounts of mis-/un-folded proteins will accumulate in the cell. In the case of K^+^ deficiency, *Pseudomonas* sp. NLX-4 launched a stress response to up-regulate expression of chaperonin (GroES) and heat shock protein (HSP10), which played an important role in protein folding (Maleki et al. 2016).

### Global expression of novel transcripts and sRNA

The novel transcripts expressed differentially among the control and treatment samples were also analyzed using the customized pipeline using edgeR, limma and Glimma packages. The RNA sequencing has resulted in total of 9681(CK-1), 9931(CK-2), 9424 (CK-3), 10,010 (Treatment-1), 11,470 (Treatment-2) and 11,695 (Treatment-3) transcripts, respectively. Out of which, 208, 229, 215, 186, 222 and 230 novel transcripts encoded for the coding regions and 9473, 9702, 9209, 9824, 11,248 and 11,465 novel transcripts encoded for the non-coding regions, respectively (Additional file 1: Fig. S18). Results obtained from the differential expression results have showed that a total of 847 novel transcript encoding genes were highly up-regulated with ≥ 2.0-fold change values and 169 novel transcript encoding genes were down regulated with ≤ 2.0-fold change values, respectively. Novel transcripts encoding for succinate dehydrogenase/fumarate reductase (TU1181), uncharacterized MFS-type transporter YbfB (TU1209), DNAJ domain (TU1447) were highly up-regulated and transcripts encoding for domain of unknown function (TU263), GGDEF domain (TU882), glucose-6-phosphate dehydrogenase (TU798), aldo/keto reductase (TU209), P-loop nucleoside triphosphate hydrolase (TU427), polyhydroxyalkanoic acid protein system (TU1525), HTH ArsR-type DNA-binding domain (TU955), thioesterase (TU1556), cytidine and deoxycytidylate deaminase (TU331), ABC-type transporter (TU1129), metal-sensitive transcriptional repressor (TU957), DNA polymerase (TU967), acyl-CoA dehydrogenase (TU1534), GNAT domain (TU315), alcohol dehydrogenase (TU1632), MarR-type HTH domain (TU710) and WD40 repeat (TU879) transcripts were down-regulated.

In the analysis of novel transcripts, succinate dehydrogenase is found out as one of the key enzymes in the pathway tricarboxylic acid cycle, which is part of carbohydrate metabolism (Araujo et al. 2012), and catalyzes the reaction of succinic acid converting to tartaric acid (Pan et al. 2010). Uncharacterized MFS-type transporter YbfB is involved in the transmembrane transport (Aminov et al. 2002), which is related to the secretion of bacterial secondary metabolites (ionophore, EPS, amino acids, and organic acids, et al.). DNAJ domain is involved in protein folding in the cell by acting as a co-molecular chaperone under environmental stresses (Bascos 2008). Therefore, combining with the analysis results of novel transcripts indicating the genes encoding succinate dehydrogenase/fumarate reductase (TU1181), uncharacterized MFS-type transporter YbfB (TU1209), DNAJ domain (TU1447) were highly up up-regulated, our opinion is that the essential elements bacteria need caused rock microbial-dissolving and the EPS, amino acids, organic acids secreted by bacterial strain accelerate rock dissolving were further confirmed.

Overall, a major disadvantage in current external-soil spray seeding technology is that the soil substrates used in these eco-restoration technologies showed poor rock interface fusion. Our present study displays a great potential to overcome these disadvantages by employing multifunctional bacteria such as *Pseudomonas* sp. NLX-4 strain to improve the interactions between the soil substrates and rock samples. Moreover, our study lays a firm foundation to further explore the deeper molecular mechanisms of rock dissolution by identifying the DEGs involved in this process.

### Limitations of the study

Although we have isolated and screened out the efficient bacterium *Pseudomonas* sp. NLX-4 that can be applied for improving the current external-soil spray seeding technology, all experiments were completed under the optimal experimental conditions. Therefore, a field experiment combining *Pseudomonas* sp. NLX-4 with external-soil spray seeding technology should be conducted to verify the effect of this strain in the natural environment of the rock mining site. Furthermore, proteome sequencing will be a valuable tool to further research and explain the relationship between genes, pathways, proteins and metabolites, so that the molecular mechanisms could be fully revealed. In any case, the role of *Pseudomonas* sp. NLX-4 and its selected genetic resources in the improvement of external-soil spray seeding technology is worth exploring.

### Supplementary Information

**Additional file 1: Figure S1.** Step-wise pipeline of experiments implemented in our study 1) rock sampling, 2) screening of bacteria, 3) isolating efficient strain, 4) identification of strain, 5) silicate rock-dissolution experiments, 6) NLX-4 strain’s effective secretory compounds, 7) NLX-4 whole-genome sequencing, 8) NLX-4 genome-wide transcriptome and 9) qRT-PCR experiment. **Table S1.** Indoleacetic acid and siderophore productions of the tested strains. **Table S2.** The contents of the NLX-4 metabolites associated with rock dissolution. **Table S3.** The contents of the bacterial metabolites produced by strain NLX-4 cultured with K-bearing rock samples and K^+^, respectively. **Figure S2.** a) Cell morphologies of strain NLX-4 (×1000); b) Neighbor-joining phylogenetic tree reconstructed based on 16S rDNA sequences, which showed the phylogenetic relationships between strain NLX-4 and related type bacteria. Bootstrap values (expressed as percentages of 1000 replications) greater than 50% are shown at branch points. The scale bars represent 0.05 substitutions per nucleotide position. **Table S4.** Data obtained from Illumina HiSeq4000 and PacBio RSII SMRT sequencing systems. **Figure S3.** The sequencing workflow applied for the Illumina HiSeq 4000 and PacBio RSII sequencing systems. **Figure S4.** The analysis workflow applied in this study for the assembly of the NLX-4 genome using hybrid analysis approach implementing the genome sequences obtained from Illumina and PacBio systems. **Figure S5.** The data analysis pipeline used for biological contextualization of the *Pseudomonas *sp NLX4 strain. **Table S5.** Lists the final results obtained from the genome assembly and genome biological contextualization. a) results obtained after the RS_HGAP (SMRT analysis suite) and Celera software pipeline, b) gene prediction using Glimmer, c) ncRNA prediction, d) tandem repeat prediction, e) CRISPR finder and f) biological contextualization (GO, COG, InterPro, KEGG, Swiss-Prot and BLAST-NR. **Table S6A.** List of primers used for qRT-PCR of NLX-4 genes which were differentially expressed in transcriptome analysis. **Table S6B.** Summary of sequences analysis of *Pseudomonas *NLX-4 genome. **Table S7.** **a)** Summary of mapping to genes; **S7b)** Summary of mapping to genomes. **Figure S6** The GC skewness of the *Pseudomonas *sp NLX-4 strain and the genome-wide distribution of different cellular process. **Figure S7.** Shows the distribution and statics of length and mass of the polymerase reads and subreads, respectively. a) The results are represented for both raw reads and clean reads, respectively; b) base composition of data. On the X axis, 1-90 bp represents the base position of read1, and 91-180 bp represents the base position of read; c) shows the reads base mass distribution. **Figure S8.** a) Correlation analysis of GC content and Depth: The abscissa is the GC content, and the ordinate is the average sequencing depth; b) shows the Kmer Analysis Graph where the abscissa is Depth, and the ordinate is the ratio of the frequency at each depth to the total frequency. Without considering the sequencing error rate, the heterozygosity and repetition of the genome. **Figure S9.** Gene Prediction glimmers. **Figure S10.** Birds eye view of the distribution of reads mapped to the reference genome, each figure shows the distribution of genes and also the distribution of reads in the longest 1 chromosome/scaffold. **Figure S11.** The results of randomness assessment showing the distribution of reads mapped to the reference genome. **Figure S12.** Distribution of gene’s coverage for individual sequenced samples. **Figure S13.** Correlation analyses of three biological replicates in treatment and CK groups, respectively. The Pearson correction coefficients are shown in the upper right corner of the plot. **Figure S14.** Detailed workflow implemented for the illumina RNA-Sequencing and the sequencing analysis pipeline for obtaining for the gene annotations. **Figure S15.** The enriched and significant KEGG pathways based on their differentially expressed genes and the total number of genes belonging to each pathway. **Figure S17.** The qRT-PCR analysis of the specifically selected six differentially expressed genes involved in the rock-dissolution process, selected based on the RNA-Seq results. **Figure S18.** The differentially expressed genes (DEGs) obtained from transcriptome analysis. a) fold change results b) DEGs both up and down-regulated c) volcano plot. **Figure S19.** a) Total number of novel transcripts discovered based on the transcriptome data analysis, which includes both novel transcripts in coding and non-coding regions; b) Total number of differentially expressed sRNA in control and treatment samples; c) distribution of the total number of sRNA’s and their length; d) Top differentially expressed sRNA’s represented by its candidate-ID along with its start and ending regions.

## Data Availability

Full genomic and transcriptomic datasets of *Pseudomonas* sp. NLX-4 are available at National Center for Biotechnology Information (NCBI) https://www.ncbi.nlm.nih.gov/geo/query/acc.cgi?acc=GSE155316 with the GEO accession number GSE155316. This study did not generate new unique reagents.
