# Supplementary material for: Genome and transcriptome analysis of rock-dissolving Pseudomonas sp. NLX-4 strain
Source: Bioresour Bioprocess. 2022 Jun 1;9(1):63. doi: 10.1186/s40643-022-00548-w (PMC10992899; doi:10.1186/s40643-022-00548-w)
Supplement: Supplementary file 1 — Additional file 1: Figure S1. Step-wise pipeline of experiments implemented in our study 1) rock sampling, 2) screening of bacteria, 3) isolating efficient strain, 4) identification of strain, 5) silicate rock-dissolution experiments, 6) NLX-4 strain’s effective secretory compounds, 7) NLX-4 whole-genome sequencing, 8) NLX-4 genome-wide transcriptome and 9) qRT-PCR experiment. Table S1. Indoleacetic acid and siderophore productions of the tested strains. Table S2. The contents of the NLX-4 metabolites associated with rock dissolution. Table S3. The contents of the bacterial metabolites produced by strain NLX-4 cultured with K-bearing rock samples and K+, respectively. Figure S2. a) Cell morphologies of strain NLX-4 (×1000); b) Neighbor-joining phylogenetic tree reconstructed based on 16S rDNA sequences, which showed the phylogenetic relationships between strain NLX-4 and related type bacteria. Bootstrap values (expressed as percentages of 1000 replications) greater than 50% are shown at branch points. The scale bars represent 0.05 substitutions per nucleotide position. Table S4. Data obtained from Illumina HiSeq4000 and PacBio RSII SMRT sequencing systems. Figure S3. The sequencing workflow applied for the Illumina HiSeq 4000 and PacBio RSII sequencing systems. Figure S4. The analysis workflow applied in this study for the assembly of the NLX-4 genome using hybrid analysis approach implementing the genome sequences obtained from Illumina and PacBio systems. Figure S5. The data analysis pipeline used for biological contextualization of the Pseudomonas sp NLX4 strain. Table S5. Lists the final results obtained from the genome assembly and genome biological contextualization. a) results obtained after the RS_HGAP (SMRT analysis suite) and Celera software pipeline, b) gene prediction using Glimmer, c) ncRNA prediction, d) tandem repeat prediction, e) CRISPR finder and f) biological contextualization (GO, COG, InterPro, KEGG, Swiss-Prot and BLAST-NR. Table S6A. List of p [file 40643_2022_548_MOESM1_ESM.docx]

**Supplementary Information**

**Genome and Transcriptome Sequencing of Novel *Pseudomonas* sp. NLX-4 Strain Involved in Bio-restoration of Over Exploited Mining Sites**

*Yanwen Wu,^1,2,5#^ Ayyappa Kumar Sista Kameshwar,^2#^ Bo Zhang,^3^ Feifei Chen,^4^ Wensheng Qin,^2^ Miaojing Meng,^1^ Jinchi Zhang^1*^*

^1^ Co-innovation Center for Sustainable Forestry in Southern China, Jiangsu Province Key Laboratory of Soil and Water Conservation and Ecological Restoration, Nanjing Forestry University, 159 Longpan Road, Nanjing, Jiangsu 210037, China.

^2^ Department of Biology, Lakehead University, 955 Oliver Road, Thunder Bay, Ontario, P7B 5E1, Canada.

^3^ Department of Biology, University of Miami, Coral Gables, FL 33124, USA.

^4^ Co-innovation Center for Sustainable Forestry in Southern China, Jiangsu Province Key Laboratory for Prevention and Management of Invasive Species, Nanjing Forestry University, 159 Longpan Road, Nanjing, Jiangsu 210037, China.

^5^ Learning Support Team, St Margaret's School, Victoria, BC, V8X 3P7, Canada (current address)

***Corresponding author:** wqin@lakeheadu.ca

**^#^**Y.W. and AKSK Contributed equally


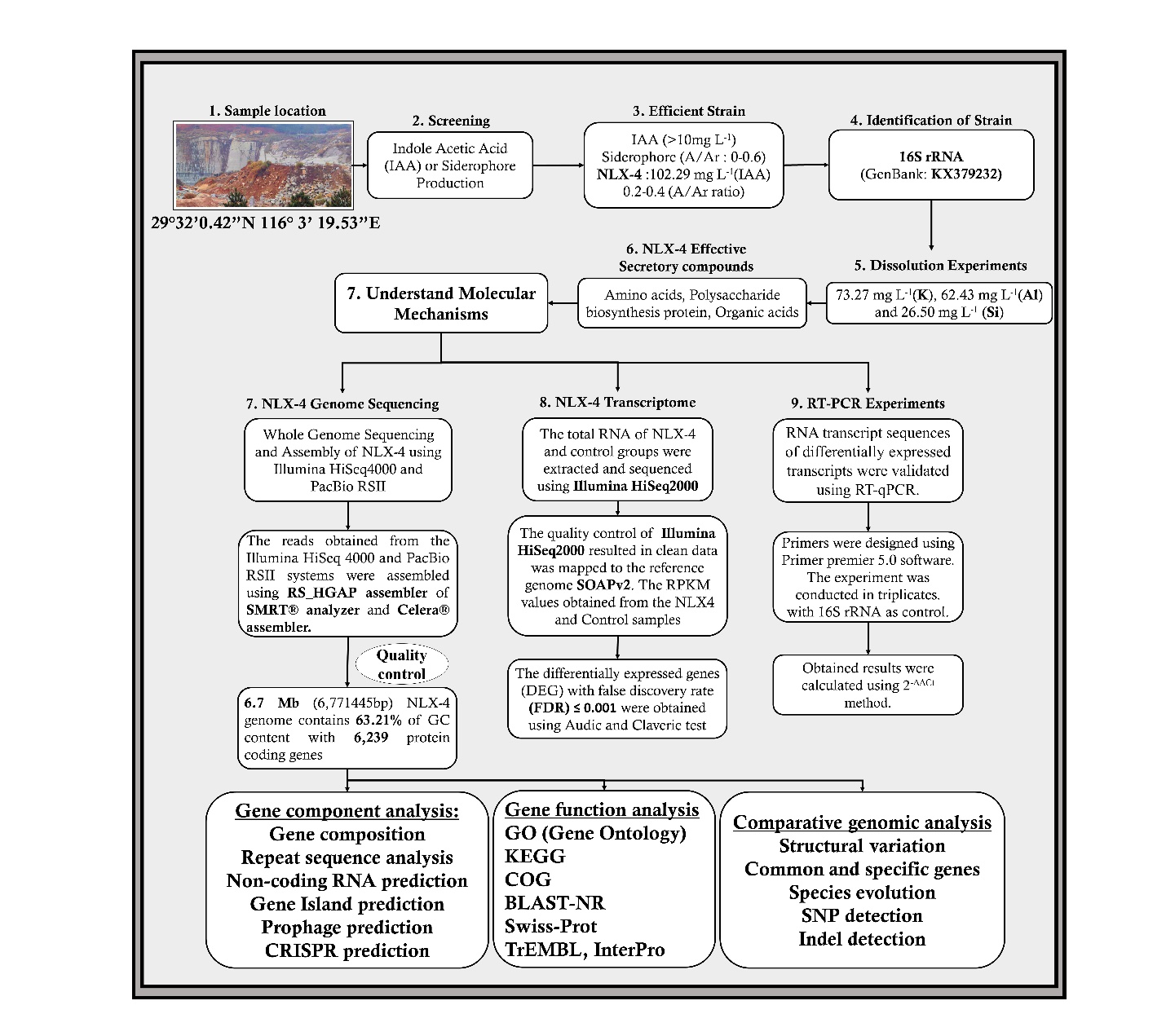


**Fig. S1** Step-wise pipeline of experiments implemented in our study 1) rock sampling, 2) screening of bacteria, 3) isolating efficient strain, 4) identification of strain, 5) silicate rock-dissolution experiments, 6) NLX-4 strain’s effective secretory compounds, 7) NLX-4 whole genome sequencing, 8) NLX-4 genome-wide transcriptome and 9) qRT-PCR experiment.

**Table S1** Indoleacetic acid and siderophore productions of the tested strains.

| **Strain** | **IAA (mg🞄L^-1^)** | **Siderophore** |
| --- | --- | --- |
| NLX-1 | 80.49 | **++++** |
| NLX-2 | 0 | **+++** |
| NLX-3 | 66.12 | **+++++** |
| **NLX-4** | **102.29** | **++++** |
| NLX-5 | 75.15 | **-** |
| NLX-6 | 42.25 | **+++++** |
| NLX-7 | 75.36 | **++++** |
| NLX-8 | 45.76 | **++++** |
| NLX-9 | 9.95 | **+++** |
| NLX-10 | 0 | **-** |
| NLX-11 | 68.37 | **-** |
| NLX-12 | 83.6 | **++++** |
| NLX-13 | 0.83 | **+** |
| NLX-14 | 106.84 | **+++** |
| NLX-15 | 0 | **+** |
| NLX-16 | 0 | **-** |
| NLX-17 | 55.92 | **+++++** |
| NLX-18 | 81.21 | **++++** |
| NLX-19 | 110.48 | **+++** |
| NLX-20 | 13.29 | **++** |
| NLX-21 | 20.36 | **-** |
| NLX-22 | 59.7 | **++++** |

**Note:** Lower A/Ar ratio represents more siderophore productions. A/Ar: 0-0.2 (+++++); 0.2-0.4 (++++); 0.4-0.6 (+++); 0.6-0.8 (++); 0.8-1.0 (+); no production (-).

**Table S2** The contents of the NLX-4 metabolites associated with rock dissolution.

| **Time**  **(days)** | **Exo-polysaccharide**  **(g L^-1^)** | **Amino acid**  **(g L^-1^)** | **Oxalic acid**  **(mg L^-1^)** | **Citric acid**  **(mg L^-1^)** | **Tartaric acid**  **(mg L^-1^)** | **Malic acid (mg L^-1^)** |
| --- | --- | --- | --- | --- | --- | --- |
| 0 | 0 | 0 | 0 | 0 | 0 | 0 |
| 2 | 3.49±0.33 | 14.14±0.49 | 41.99±1.54 | 31.15±1.30 | 364.19±2.63 | 38.88±1.91 |
| 5 | 6.88±0.20 | 24.06±1.86 | 65.70±2.45 | 47.84±1.63 | 569.41±10.40 | 74.15±1.05 |
| 9 | 8.21±0.22 | 35.25±1.98 | 78.20±2.58 | 64.22±2.37 | 690.08±7.26 | 91.68±2.00 |
| 12 | 9.09±0.25 | 43.96±1.87 | 70.57±3.01 | 59.45±0.80 | 777.38±8.71 | 123.68±6.43 |
| 15 | 12.12±0.78 | 74.70±2.60 | 49.57±2.42 | 44.77±0.72 | 956.98±5.53 | 103.32±2.69 |
| 22 | 15.45±1.02 | 87.74±1.95 | 31.88±1.14 | 35.99±1.05 | 992.32±5.51 | 84.73±1.36 |
| 30 | 13.54±0.14 | 97.47±4.10 | 26.08±1.19 | 32.44±1.11 | 1025.87±5.14 | 75.58±3.51 |

**Table S3** The contents of the bacterial metabolites produced by strain NLX-4 cultured with K-bearing rock samples and K^+^, respectively.

| **Effective secretory compounds** | **Treatment** | |
| --- | --- | --- |
|  | **NLX-4+rock** | **NLX-4+K^+^(CK)** |
| Polysaccharide (g L^-1^) | 12.12±0.78*** | 3.22±0.20 |
| Amino acid (g L^-1^) | 74.70±2.60*** | 48.63±2.85 |
| Oxalic acid (mg L^-1^) | 49.57±2.42*** | 30.29±2.70 |
| Citric acid (mg L^-1^) | 44.77±0.72*** | 26.91±1.31 |
| Tartaric acid (mg L^-1^) | 956.98±5.53*** | 317.12±4.45 |
| Malic acid (mg L^-1^) | 103.32±2.69*** | 42.65±3.13 |

**Note:** ***indicates difference is significant at the 0.001 level.


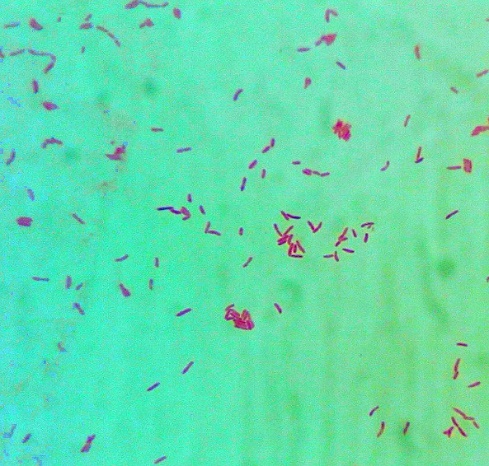


**a**

**b**


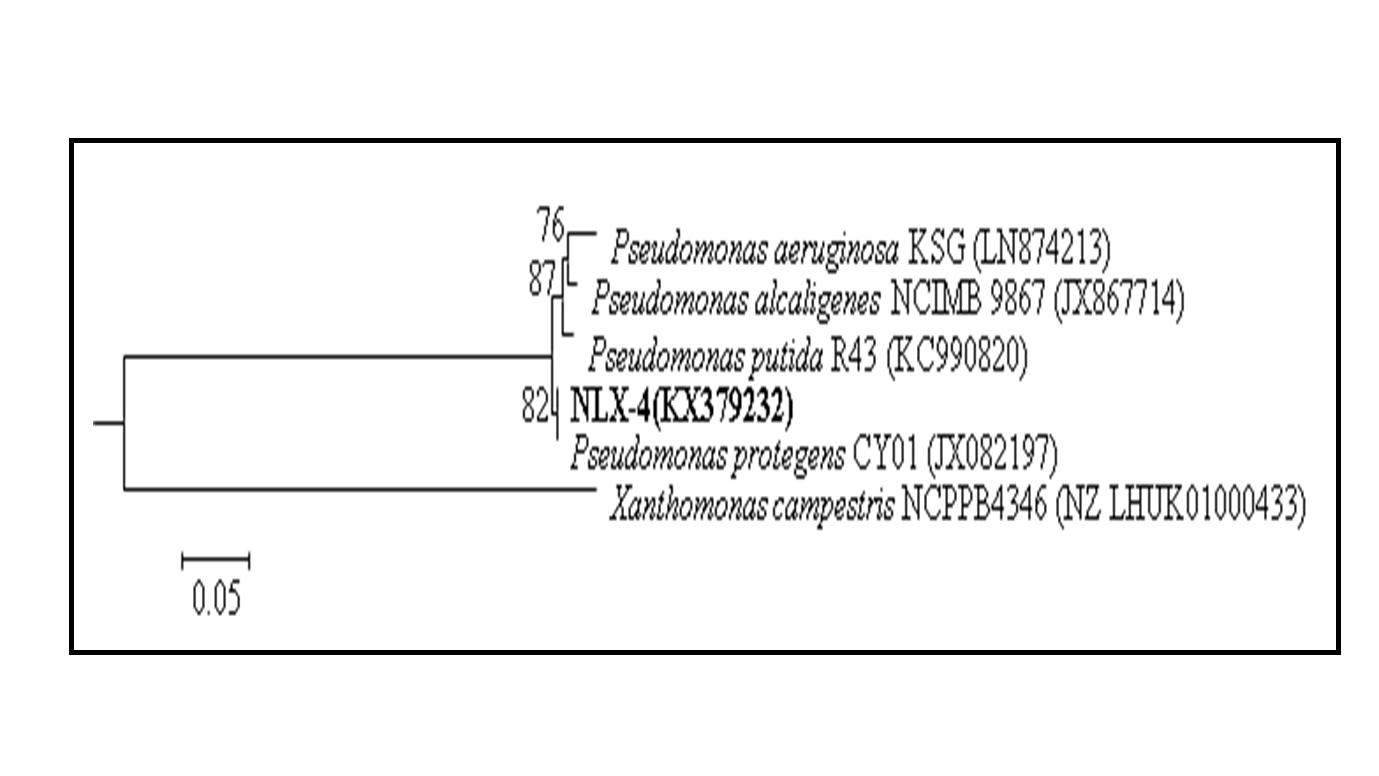


**Fig. S2** a) Cell morphologies of strain NLX-4 (×1000); b) Neighbor-joining phylogenetic tree reconstructed based on 16S rDNA sequences, which showed the phylogenetic relationships between strain NLX-4 and related type bacteria. Bootstrap values (expressed as percentages of 1000 replications) greater than 50% are shown at branch points. The scale bars represent 0.05 substitutions per nucleotide position.

**Table S4** Data obtained from Illumina HiSeq4000 and PacBio RSII SMRT sequencing systems.

| **Illumina HiSeq4000** | | **PacBio RSII SMRT** | |
| --- | --- | --- | --- |
| **Sample Name** | **NLX-4** | **Sample Name** | **NLX-4** |
| Insert Size(bp) | 300 | Polymerase Reads Number | 49,861 |
| Reads Length(bp) | (100:100) | Polymerase Reads Mean Length(bp) | 14,374 |
| Raw Data(Mb) | 904 | Polymerase Reads Bases(bp) | 766,172,465 |
| Adapter (%) | 0.23 | Polymerase Reads Quality | 0.85 |
| Duplication (%) | 8.03 | Subreads Number | 79,379 |
| Total Reads | 9,049,948 | Subreads Mean Length(bp) | 8,991 |
| Filtered Reads (%) | 9.68 | Subreads Bases(bp) | 713,706,632 |
| Low Quality Filtered Reads (%) | 1.39 | Subreads Quality | 0.85 |
| Clean Data(Mb) | 817 | Utilization Ratio | 0.95 |

**
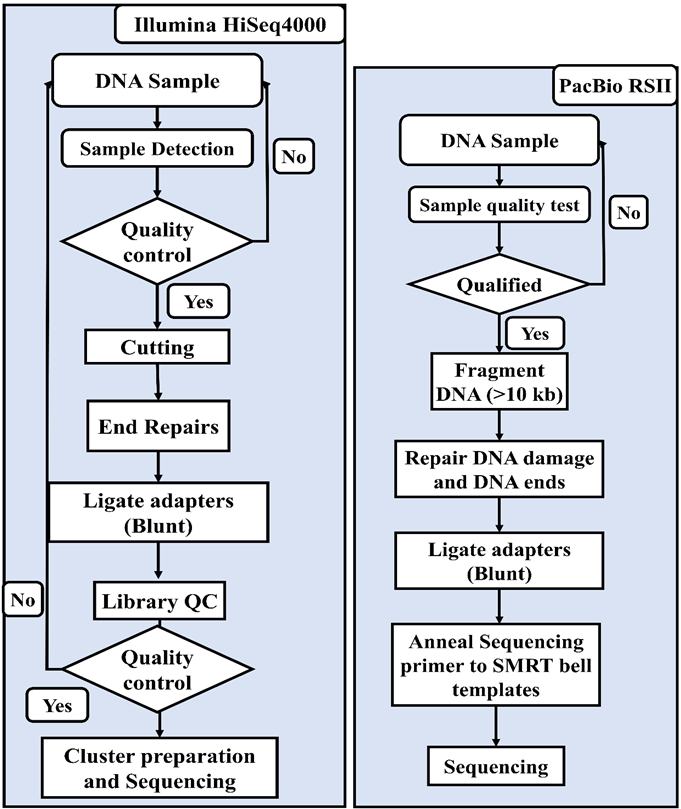
**

**Fig. S3** The sequencing workflow applied for the Illumina HiSeq 4000 and PacBio RSII sequencing systems.


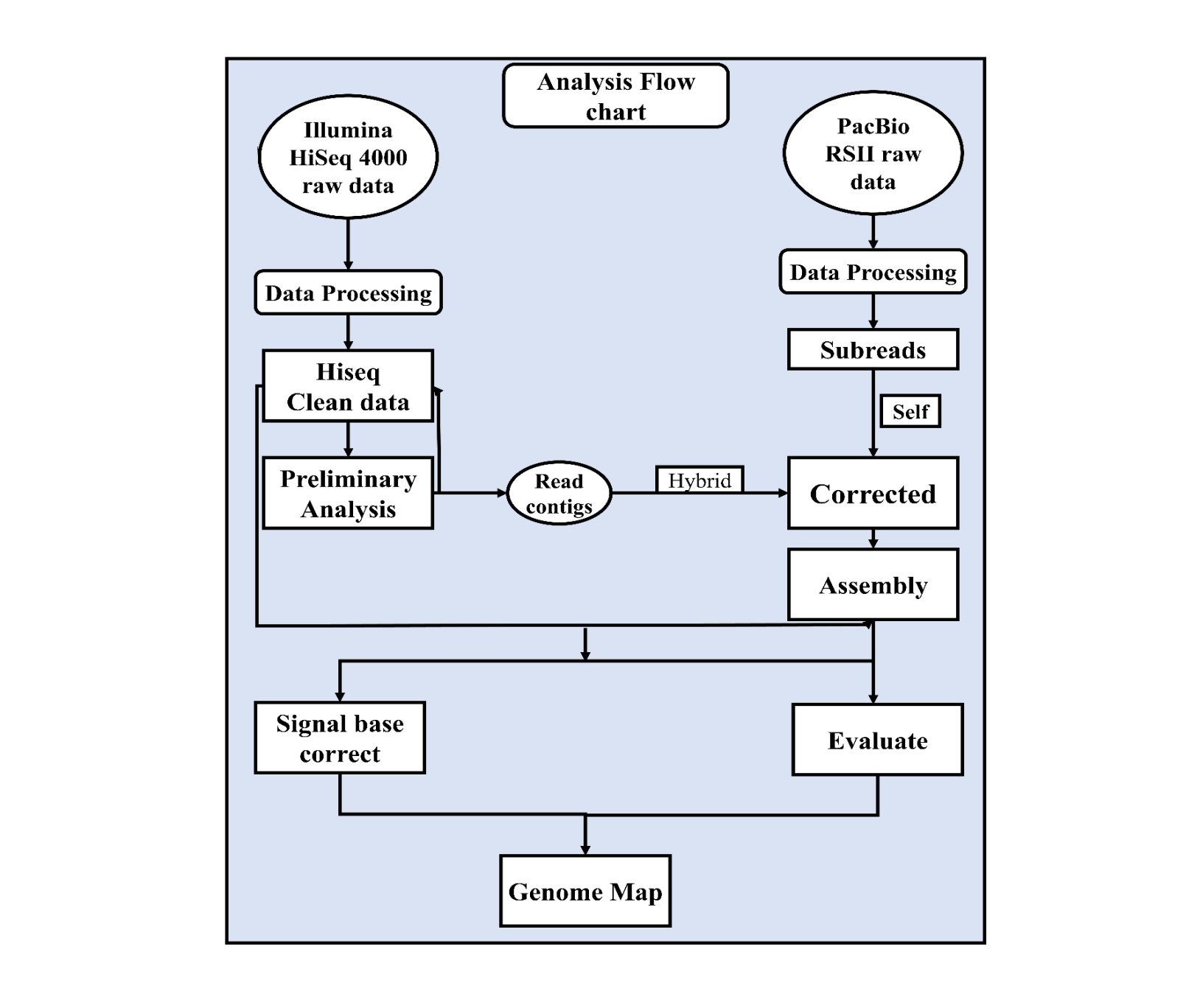


**Fig. S4** The analysis workflow applied in this study for the assembly of the NLX-4 genome using hybrid analysis approach implementing the genome sequences obtained from Illumina and PacBio systems.


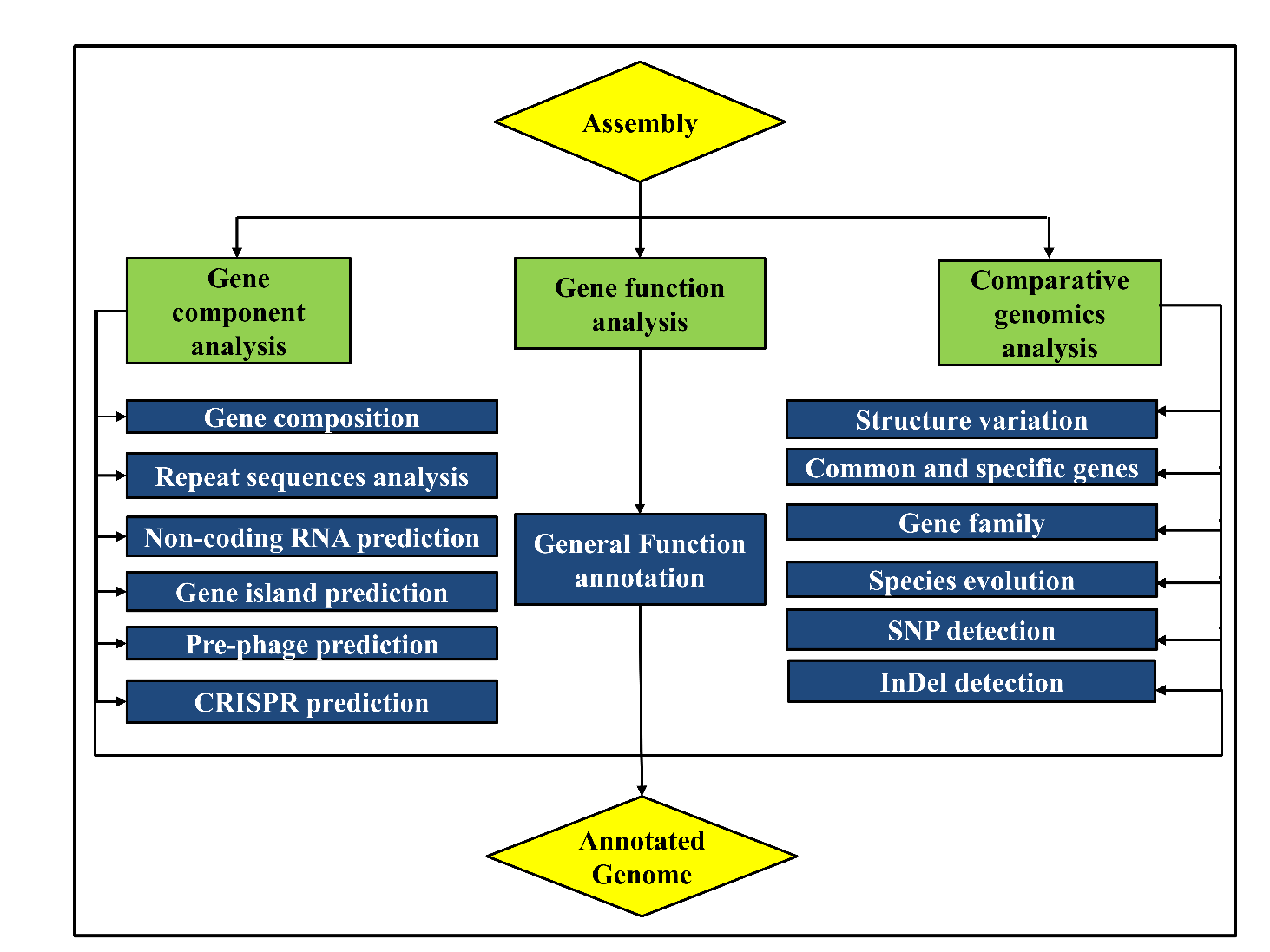
**Fig. S5** The data analysis pipeline used for biological contextualization of the *Pseudomonas* sp NLX4 strain.

**Table S5** Lists the final results obtained from the genome assembly and genome biological contextualization. a) results obtained after the RS_HGAP (SMRT analysis suite) and Celera software pipeline, b) gene prediction using Glimmer, c) ncRNA prediction, d) tandem repeat prediction, e) CRISPR finder and f) biological contextualization (GO, COG, InterPro, KEGG, Swiss-Prot and BLAST-NR.

| **Assembly Results**  **a** | |
| --- | --- |
| **Assembling result evaluation file** | |
| **Sample Name** | **NLX-4** |
| Single Base Quality (#) | 1 |
| Structure Base Quality (#) | 0.9975 |
| Reads Usage Percent (#) | 0.9915 |
| Conclusion | Yes |
| **Assembly result statistics file** | |
| **Sample Name** | **NLX-4** |
| Chromosome Number (#) | 1 |
| Total Length (bp) | 6,771,445 |
| N50 Length (bp) | 6,771,445 |
| N90 Length (bp) | 6,771,445 |
| Max Length (bp) | 6,771,445 |
| Min Length (bp) | 6,771,445 |
| GC Content (%) | 63.21 |
| ID-Circular/Linear-Length | Chromosome_1; circular:6771445 |
| **K-mer result statistics file** | |
| **Sample-name** | **NLX-4** |
| K-mer (#) | 15 |
| Kmer_Num(Mbp) | 228.57 |
| Pk_Depth (#) | 35 |
| Genome Size(Mbp) | 6.52 |
| Genome Depth (#) | 40.75 |
| **Repeat result statistic file** | |
| **Sample Name** | **NLX-4** |
| Genome Size (#) | 6,771,445 |
| GC (%) | 63.21 |
| Repeat Number (#) | 1,049 |
| Repeat Length (#) | 130,540 |
| Min Length (#) | 25 |
| Max Length (#) | 5,847 |
| Average Length (#) | 124.44 |
| Repeat Rate (%) | 1.93 |

| **Results from Glimmer** | |
| --- | --- |
| **Sample Name** | **NLX-4** |
| Gene Number | 6,239 |
| Gene Length | 5,918,817 |
| GC Content | 64.17 |
| % of Genome(Genes) | 87.41 |
| Gene Average Length | 949 |
| Gene Internal Length | 852,628 |
| Gene Internal GC Content | 56.55 |
| % of Genome(internal) | 12.59 |

**b**

| **Method** | **Type** | **Number#** | **Avg_Len** | **Total_Len** | **% in Genome** |
| --- | --- | --- | --- | --- | --- |
| TRNA-NA-Scan | tRNA | 72 | 77 | 5,612 | 0.0829 |
| rRNA_denovo | 5s | 7 | 116 | 812 | -NA- |
| rRNA_denovo | 16s | 6 | 1,527 | 9,162 | 0.4034 |
| rRNA_denovo | 23s | 6 | 2,890 | 17,340 | -NA- |
| rRNA_ho | 5S | -NA- | -NA- | -NA- | -NA- |
| rRNA_ho | 16S | -NA- | -NA- | -NA- | -NA- |
| rRNA_ho | 23S | -NA- | -NA- | -NA- | -NA- |
| RNAmmer | sRNA | 17 | 172 | 2,927 | 0.0432 |

**c**

**d**

| **Type** | **Number (#)** | **Repeat Size(bp)** | **Total Length(bp)** | **In Genome (%)** |
| --- | --- | --- | --- | --- |
| TRF | 192 | 6-1,047 | 35,083 | 0.5181 |
| Minisatellite DNA | 90 | 15-63 | 4,597 | 0.0679 |
| Microsatellite DNA  **e** | 36 | 10-6 | 1,380 | 0.0204 |

| Chr-1 Crispr1 | GGCTGGCGGTTTCGCCTTTACGGCGAGTGGGAC |
| --- | --- |
| Chr-1 Crispr2 | CCGTGCCACTCGCTGTAAAAGCGAAACCGCCAGC |
| Chr-1 Crispr3 | GTCAAACTCGCCACAAAGGCGAAACCGCCAGCGGCCACAC |
| Chr-1 Crispr4 | CTATCCCGGCCCGACATGGGCCCTGTAGCCCCCTGCGTCTCCTGCGGA |

**f**

| **Sample** | **NLX-4** |
| --- | --- |
| Total | 6239 |
| COG | 5045(80.86%) |
| GO | 3996(64.04%) |
| IPR | 5342(85.62%) |
| KEGG | 4386(70.29%) |
| NR | 6038(96.77%) |
| Swiss Prot | 3897(62.46%) |
| Over all | 6041(96.82%) |

**Table S6A:**  List of primers used for qRT-PCR of NLX-4 genes which were differentially expressed in transcriptome analysis.

| **Gene ID &Description** | **Sequence 5’-3’** | |
| --- | --- | --- |
|  | **Forward primer** | **Reverse primer** |
| **NZ1GL005331**  Amino acid-specific outer membrane pore | AAGTTCCGCGTGTCCAACAC | TCAAAGCTCGAAGGCAGTGC |
| **NZ1GL001246**  Nitric oxide dioxygenase | ATCAAAGCCACTGTGCCCCT | GTTGAACAGCGGACGCACTT |
| **NZ1GL001295**  Glutathione S-transferase | CAGGTCAAGCTGGAGCAGGA | GGCTGTCGTTGATCACCACC |
| **NZ1GL001417**  Bacterioferritin | GCATCCTCTTCCTCGAAGGC | GTGCGTTCGATACGCAGGTC |
| **NZ1GL001889**  Polysaccharide biosynthesis protein | GCATGTACCGATGGTGGAGC | TTGGCTACTCCGGACTGCAG |
| **16S rRNA** | AACCGCGAGGTGGAGCTAAT | GCGACATTCTGATTCGCGAT |

**Table S6B:** Summary of sequences analysis of *Pseudomonas* NLX-4 genome

| **Description** | **Number** |
| --- | --- |
| Insert Size (bp) | 300 |
| Reads Length (bp) | (100:100) |
| Raw Data (Mb) | 904 |
| Adapter (%) | 0.23 |
| Duplication (%) | 8.03 |
| Total Reads | 9,049,948 |
| Filtered Reads (%) | 9.68 |
| Low Quality Filtered Reads (%) | 1.39 |
| Clean Data (Mb) | 817 |
| **After Assembly** | |
| Genome Size | 6,771,445 |
| GC (%) | 63.21 |
| Repeat Number | 1,049 |
| Repeat Length | 130,540 |
| Min Length | 25 |
| Max Length | 5,847 |
| Average Length | 124.44 |
| Repeat Rate | 1.93 |

**Table S7a)** Summary of mapping to genes; **S7b)** Summary of mapping to genomes

| **a. Gene** | | | | | | | | | |
| --- | --- | --- | --- | --- | --- | --- | --- | --- | --- |
| **Sample** | | **Total reads** | **Total base pairs** | **Total mapped reads** | **Perfect match** | **<=5bp mismatch** | **Unique match** | **Multi-position match** | **Total unmapped reads** |
| **Treatment 1** | **reads number** | 24990982 | 2499098200 | 10697962 | 6828765 | 3869197 | 10642255 | 55707 | 14293020 |
|  | **percentage** | 100.00% | 100.00% | 42.81% | 27.32% | 15.48% | 42.58% | 0.22% | 57.19% |
| **Treatment 2** | **reads number** | 24662260 | 2466226000 | 6015324 | 3855412 | 2159912 | 5968251 | 47073 | 18646936 |
|  | **percentage** | 100.00% | 100.00% | 24.39% | 15.63% | 8.76% | 24.20% | 0.19% | 75.61% |
| **Treatment 3** | **reads number** | 23053138 | 2305313800 | 4651007 | 2865703 | 1785304 | 4616782 | 34225 | 18402131 |
|  | **percentage** | 100.00% | 100.00% | 20.18% | 12.43% | 7.74% | 20.03% | 0.15% | 79.82% |
| **CK 1** | **reads number** | 23478916 | 2347891600 | 9928596 | 6315556 | 3613040 | 9883855 | 44741 | 13550320 |
|  | **percentage** | 100.00% | 100.00% | 42.29% | 26.90% | 15.39% | 42.10% | 0.19% | 57.71% |
| **CK 2** | **reads number** | 23681178 | 2368117800 | 8357772 | 5383694 | 2974078 | 8323882 | 33890 | 15323406 |
|  | **percentage** | 100.00% | 100.00% | 35.29% | 22.73% | 12.56% | 35.15% | 0.14% | 64.71% |
| **CK 3** | **reads number** | 23458688 | 2345868800 | 9011855 | 5664846 | 3347009 | 8970619 | 41236 | 14446833 |
|  | **percentage** | 100.00% | 100.00% | 38.42% | 24.15% | 14.27% | 38.24% | 0.18% | 61.58% |

| **b. Genome** | | | | | | | | | |
| --- | --- | --- | --- | --- | --- | --- | --- | --- | --- |
| **Sample** | | **Total reads** | **Total base pairs** | **Total mapped reads** | **Perfect match** | **<=5bp mismatch** | **Unique match** | **Multi-position match** | **Total unmapped reads** |
| **Treatment 1** | **reads number** | 24990982 | 2499098200 | 24344932 | 15806518 | 8538414 | 24228961 | 115971 | 646050 |
|  | **percentage** | 100.00% | 100.00% | 97.41% | 63.25% | 34.17% | 96.95% | 0.46% | 2.59% |
| **Treatment 2** | **reads number** | 24662260 | 2466226000 | 22413096 | 14736337 | 7676759 | 22119377 | 293719 | 2249164 |
|  | **percentage** | 100.00% | 100.00% | 90.88% | 59.75% | 31.13% | 89.69% | 1.19% | 9.12% |
| **Treatment 3** | **reads number** | 23053138 | 2305313800 | 20972730 | 13202897 | 7769833 | 20856681 | 116049 | 2080408 |
|  | **percentage** | 100.00% | 100.00% | 90.98% | 57.27% | 33.70% | 90.47% | 0.50% | 9.02% |
| **CK 1** | **reads number** | 23478916 | 2347891600 | 22549111 | 14560281 | 7988830 | 22189053 | 360058 | 929805 |
|  | **percentage** | 100.00% | 100.00% | 96.04% | 62.01% | 34.03% | 94.51% | 1.53% | 3.96% |


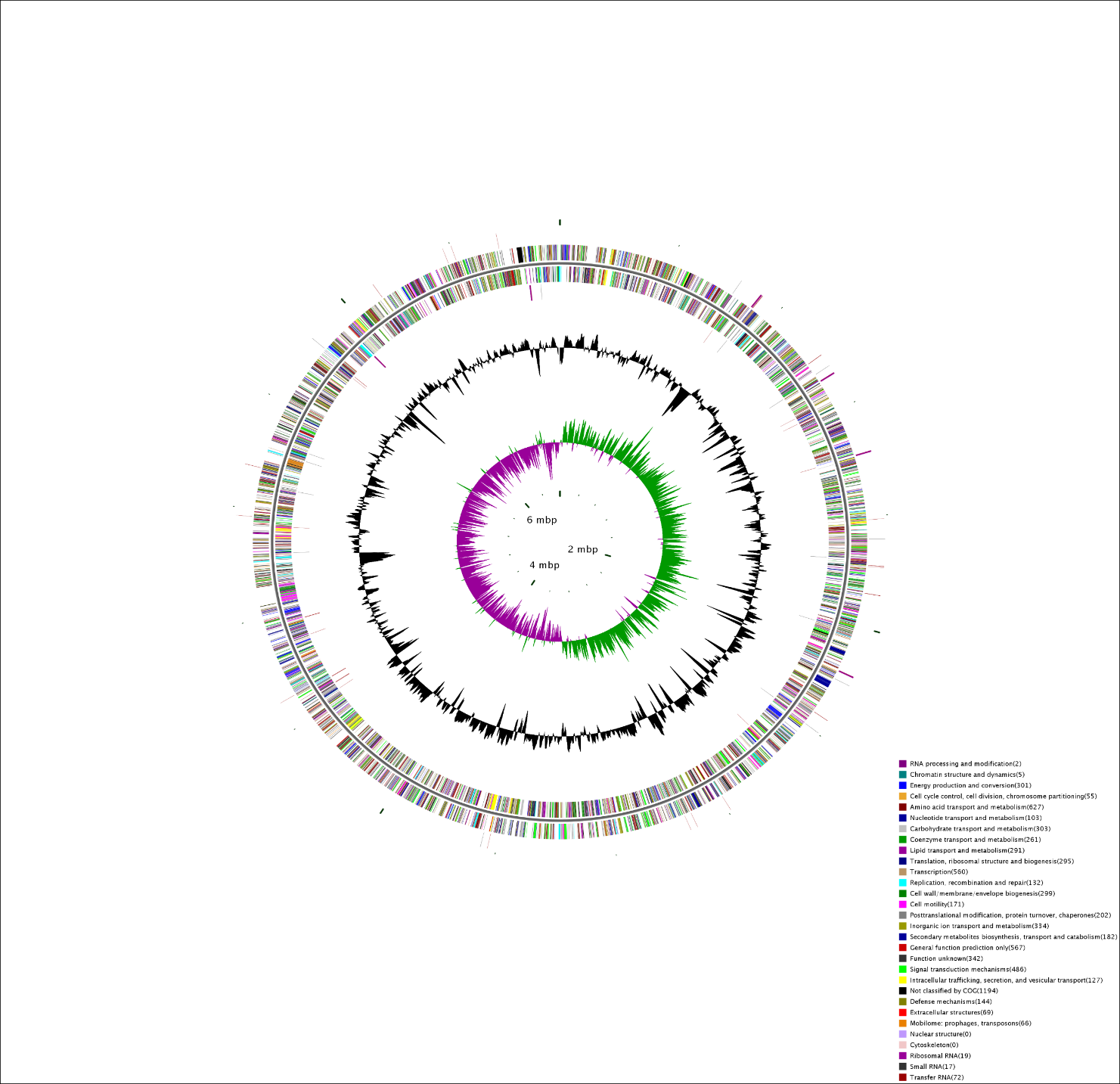

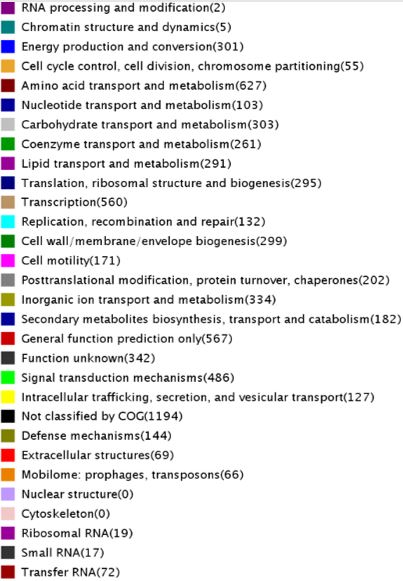


**Fig. S6** The GC skewness of the *Pseudomonas* sp NLX-4 strain and the genome wide distribution of different cellular process.

**c**

**b**

**a**


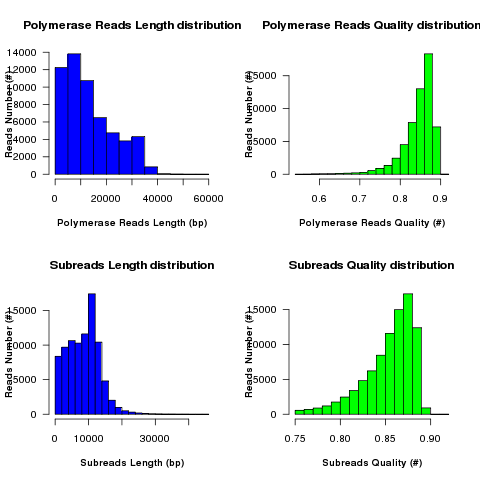


**Raw reads**


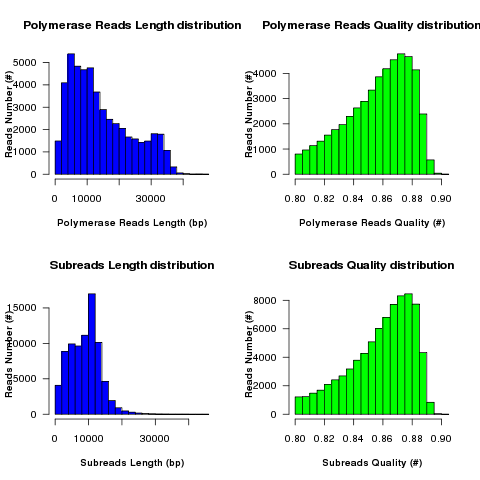


**Clean reads**


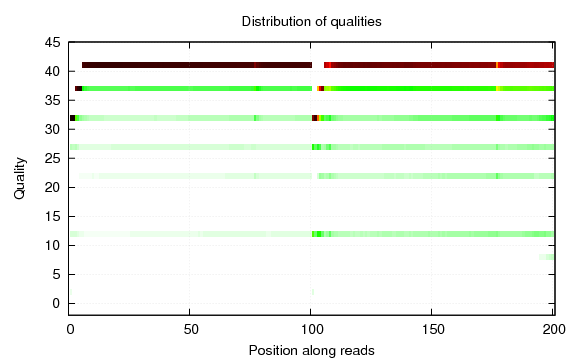

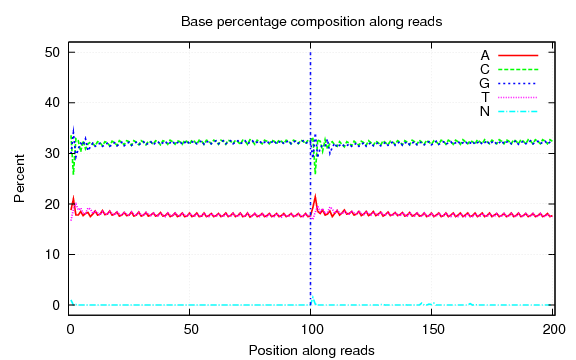


**Fig. S7** Shows the distribution and statics of length and mass of the polymerase reads and subreads respectively. a) The results are represented for both raw reads and clean reads respectively; b) base composition of data. On the X axis, 1-90 bp represents the base position of read1, and 91-180 bp represents the base position of read; c) shows the reads base mass distribution.

**Fig. S8** a) Correlation analysis of GC content and Depth: The abscissa is the GC content, and the ordinate is the average sequencing depth; b) shows the Kmer Analysis Graph where the abscissa is Depth, and the ordinate is the ratio of the frequency at each depth to the total frequency. Without considering the sequencing error rate, the heterozygosity and repetition of the genome.


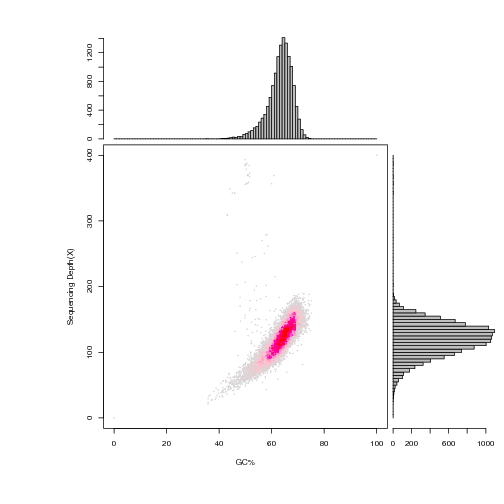

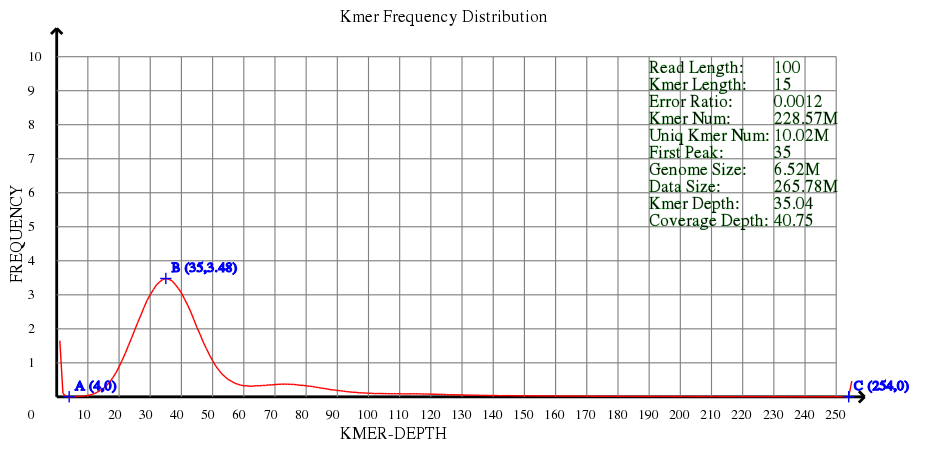


**b**

**a**


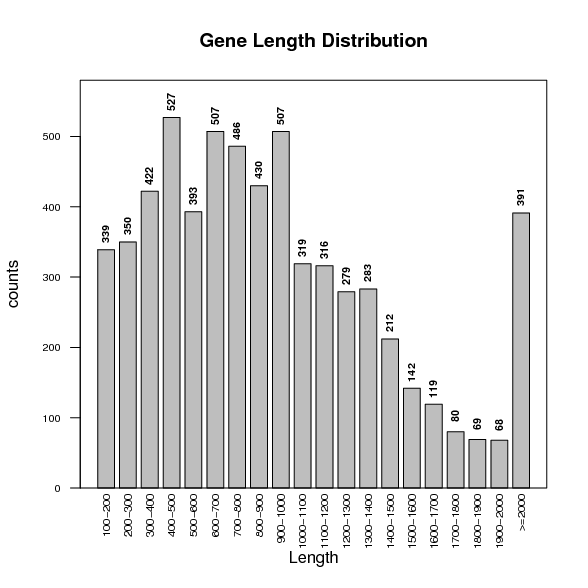


**Fig. S9** Gene Prediction glimmers.


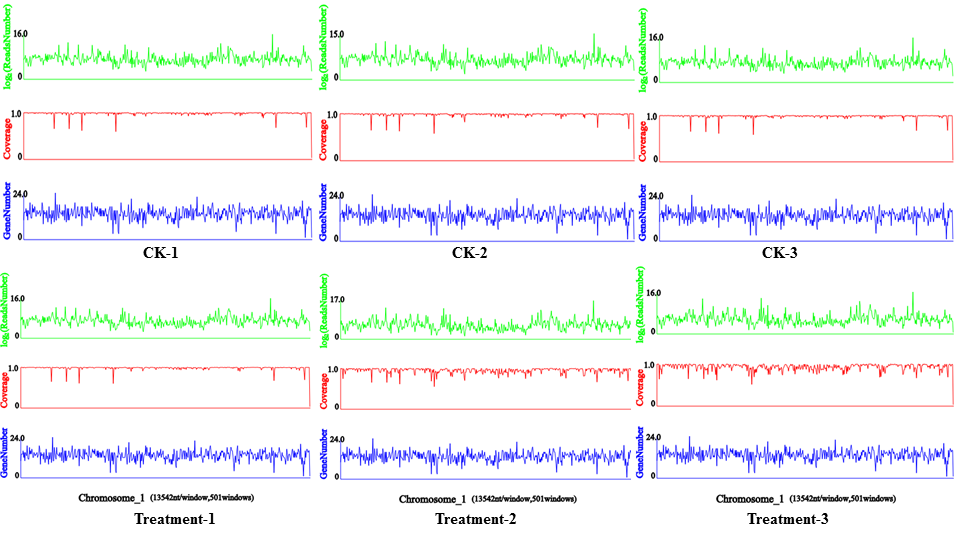
**Fig. S10** Birds eye view of the distribution of reads mapped to the reference genome, each figure shows the distribution of genes and also the distribution of reads in the longest 1 chromosome/scaffold.

**
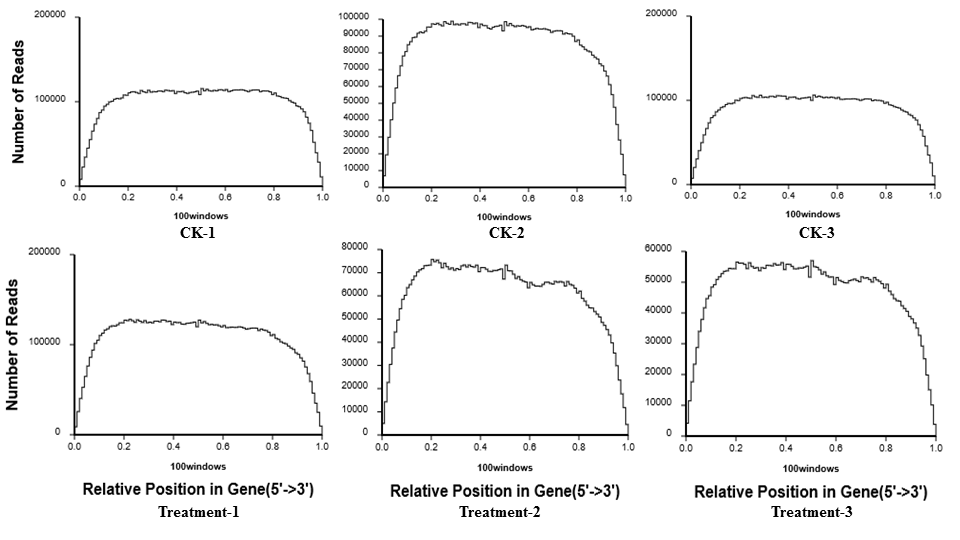
Fig. S11** The results of randomness assessment showing the distribution of reads mapped to the reference genome.


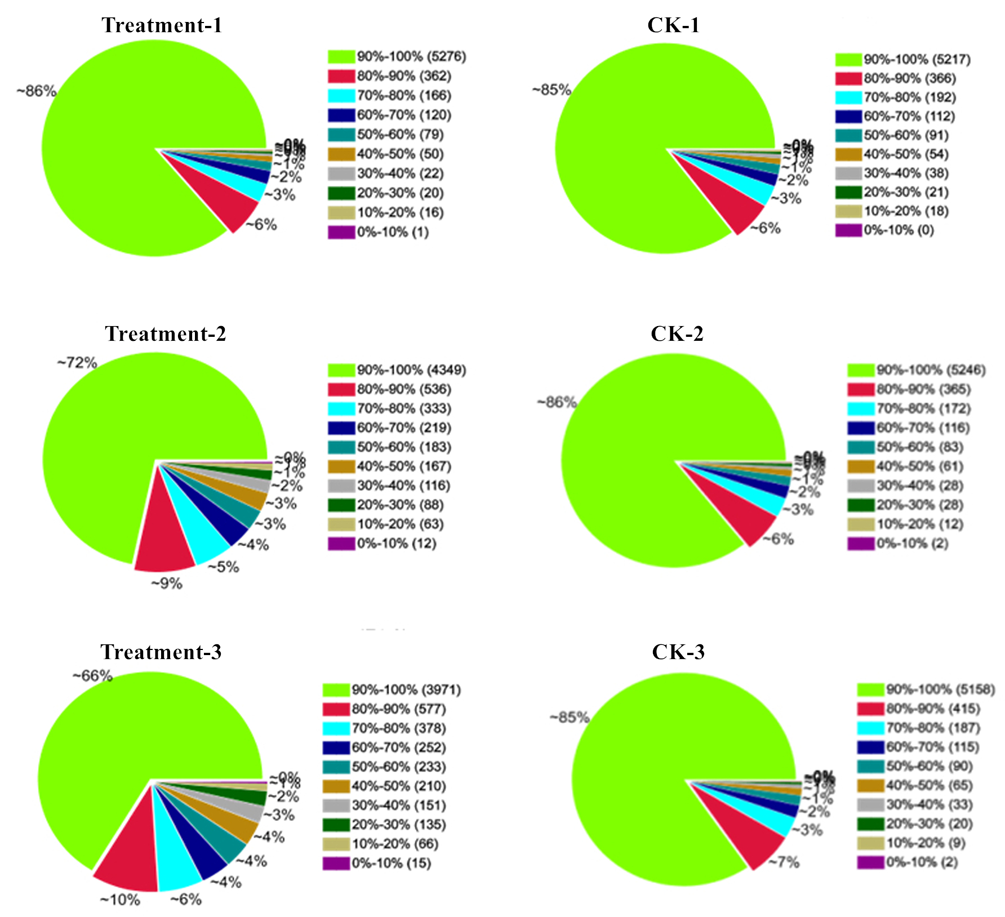
**Fig. S12** Distribution of gene’s coverage for individual sequenced samples.


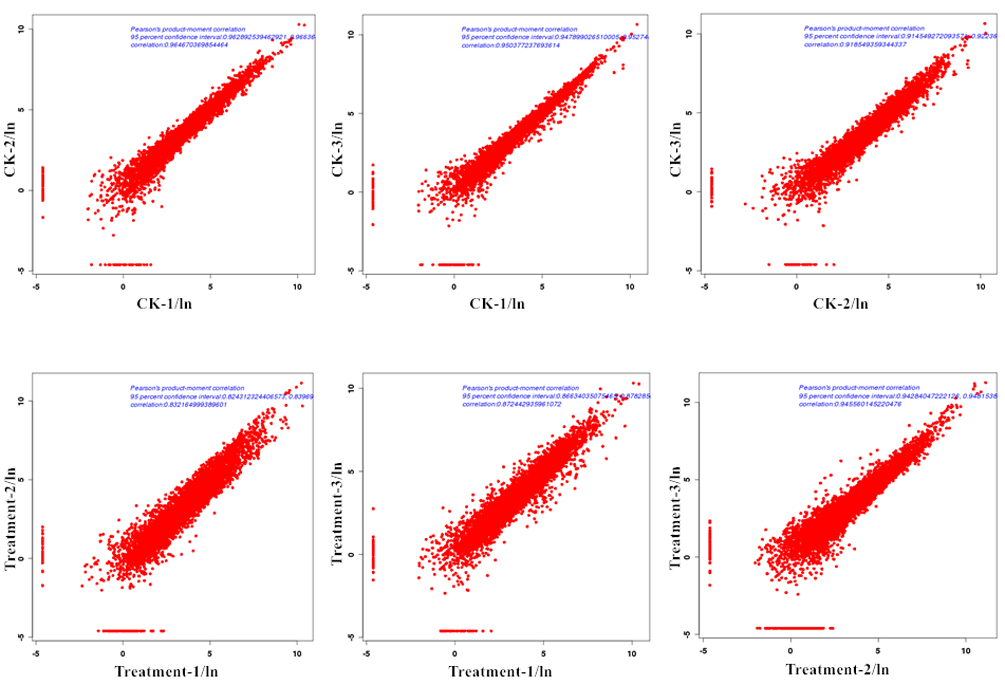


**Fig. S13** Correlation analyses of three biological replicates in treatment and CK groups, respectively. The Pearson correction coefficients are shown in the upper right corner of the plot.

**Fig. S14** Detailed workflow implemented for the illumina RNA-Sequencing and the sequencing analysis pipeline for obtaining for the gene annotations.


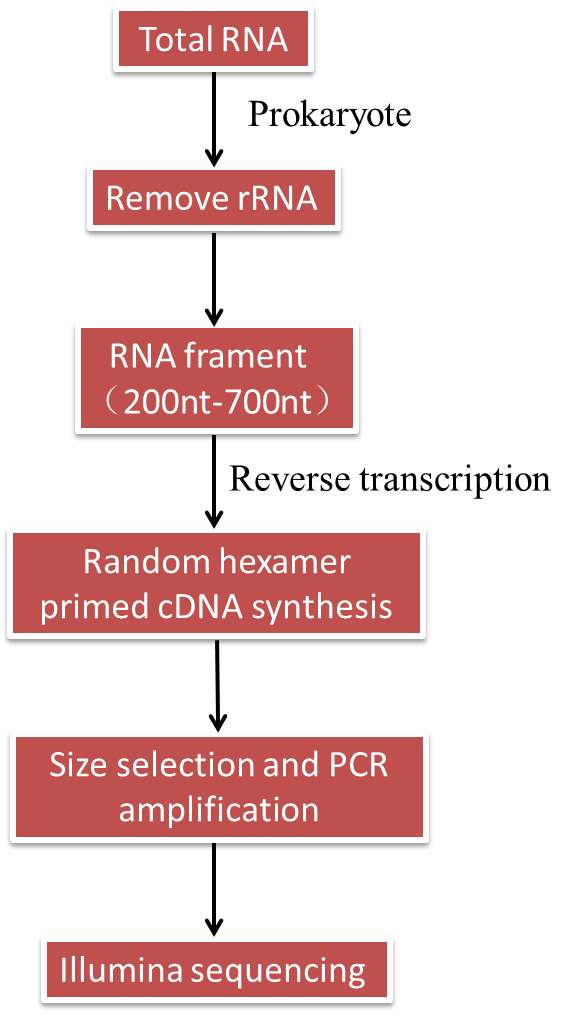

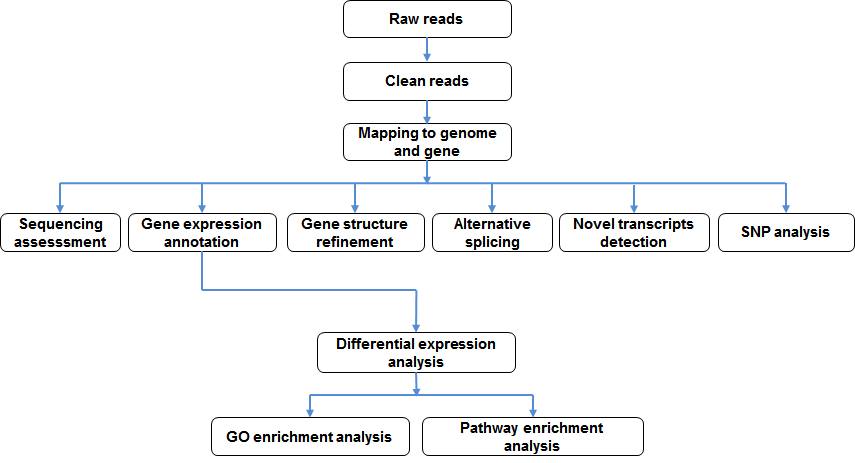

**Continued…**

**Fig. S15** The enriched and significant KEGG pathways based on their differentially expressed genes and the total number of genes belonging to each pathway.


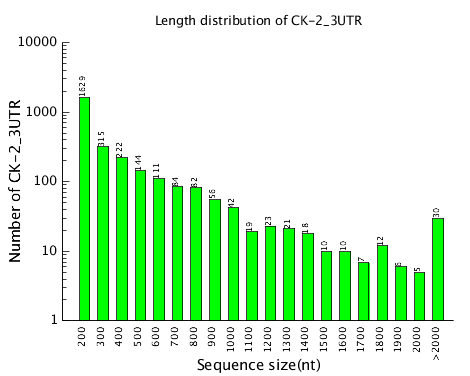

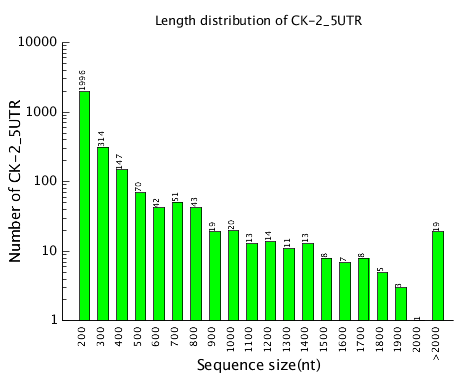

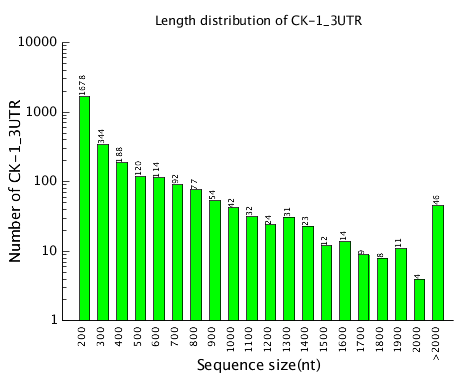

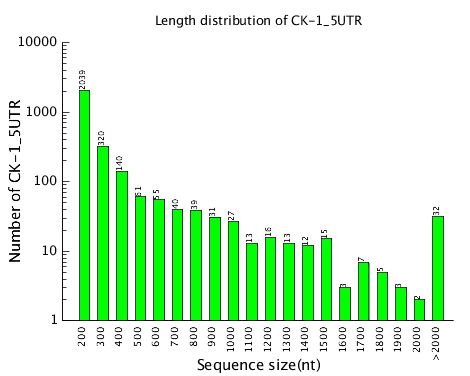

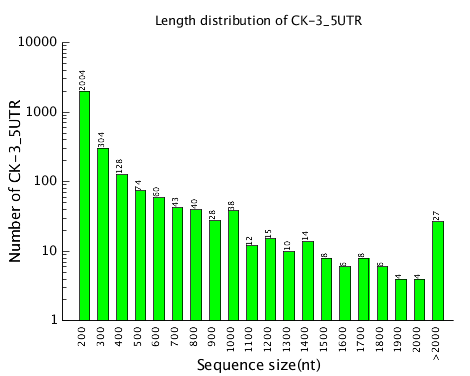

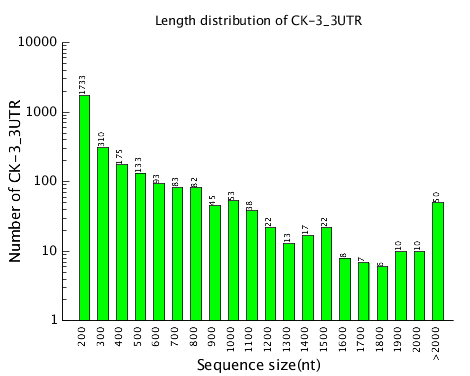


**Fig. S16** Shows the distribution of 3’ and 5’ UTR regions in the sequenced samples (control and NLX-4 samples.


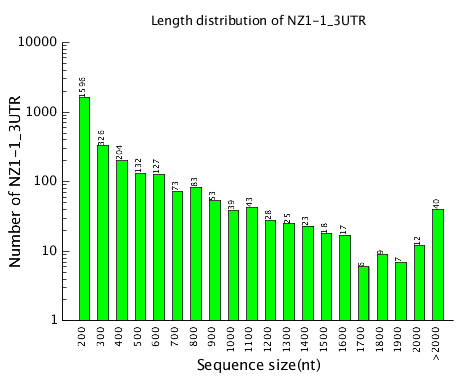

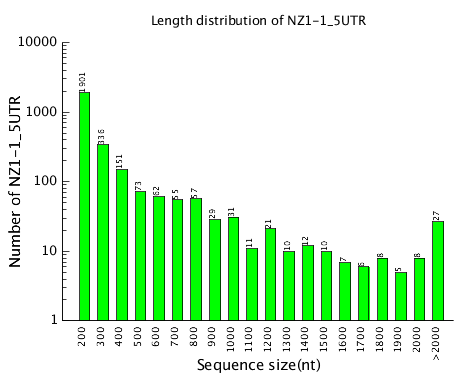

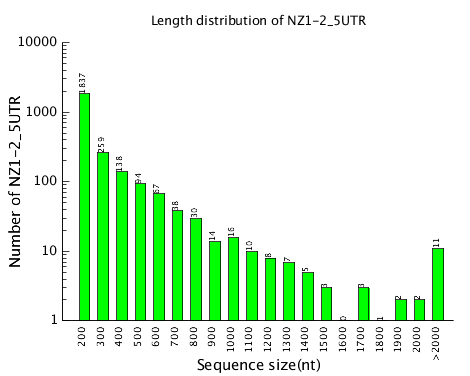

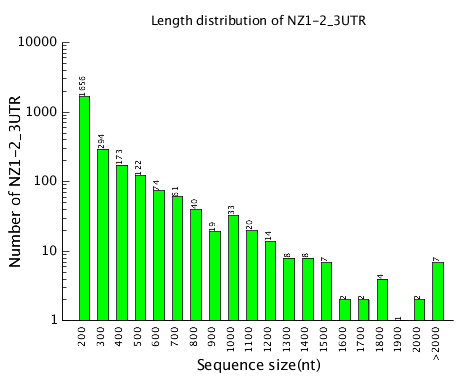

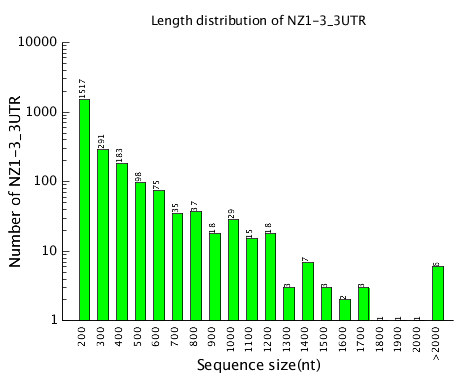

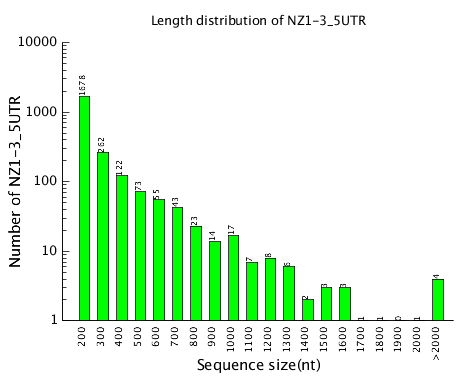

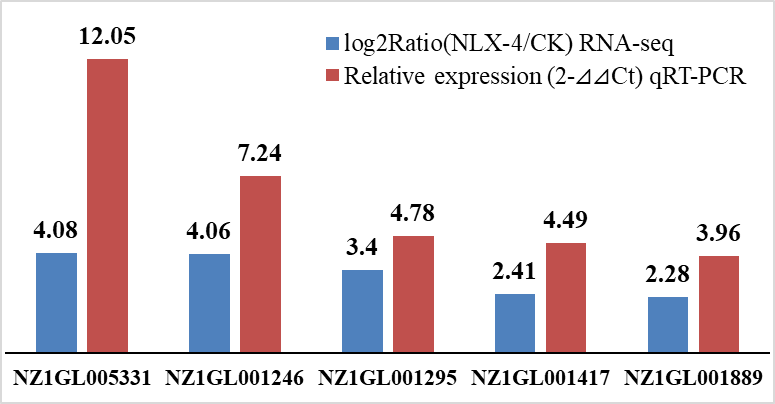

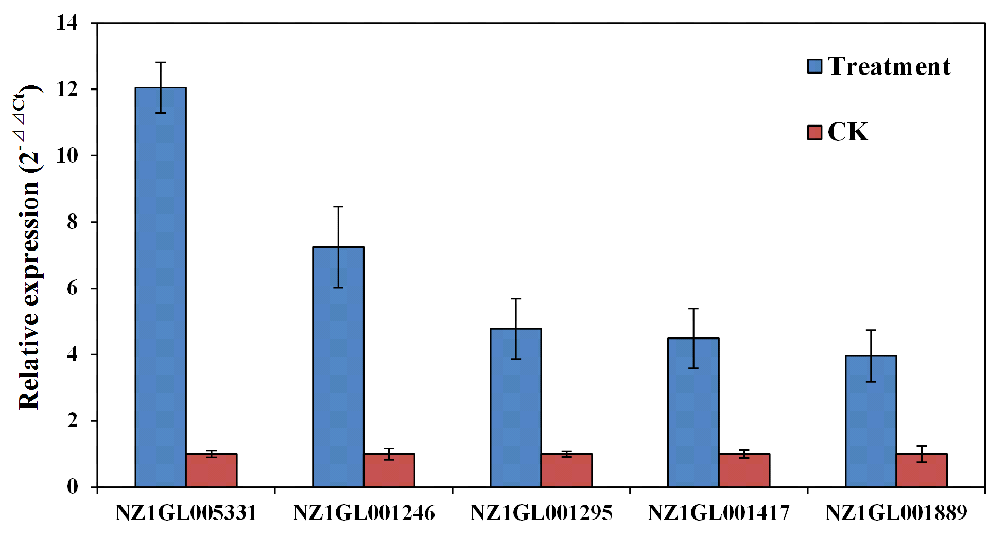


**Fig. S17** The qRT-PCR analysis of the specifically selected six differentially expressed genes involved in the rock-dissolution process, selected based on the RNA-Seq results.


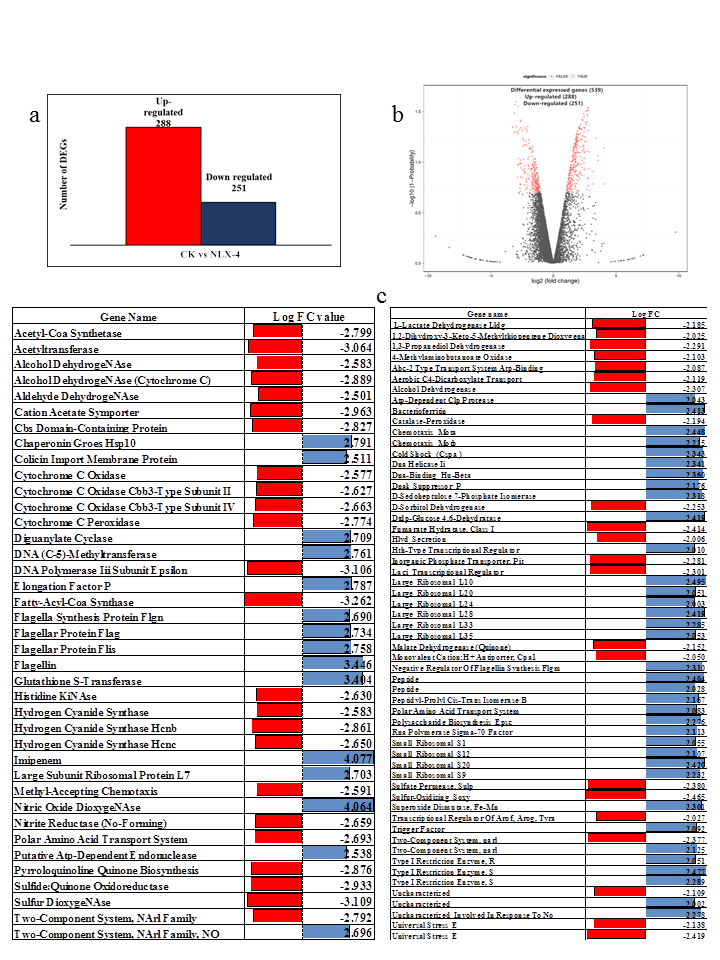


**Fig. S18** The differentially expressed genes (DEGs) obtained from transcriptome analysis. a) fold change results b) DEGs both up and down regulated c) volcano plot.

**a**

**c**

**b**


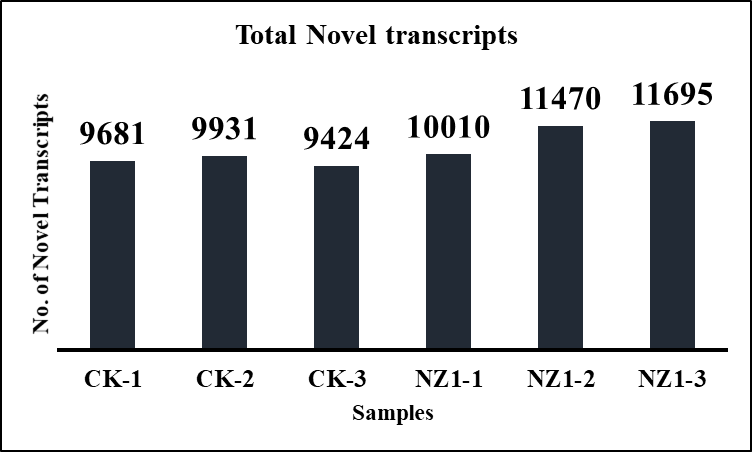

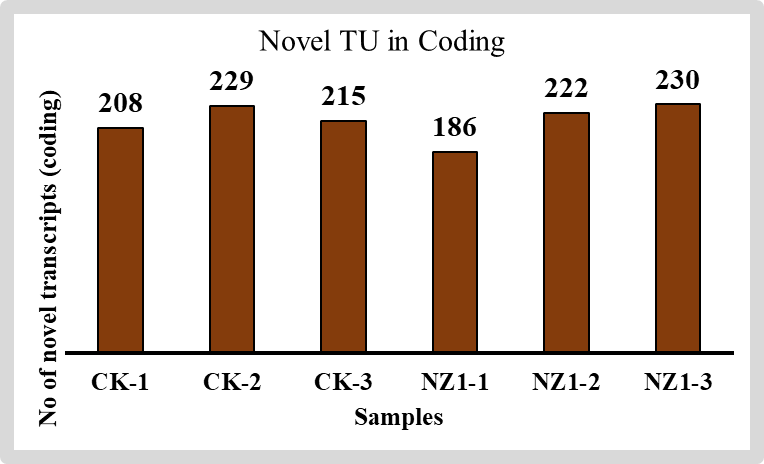

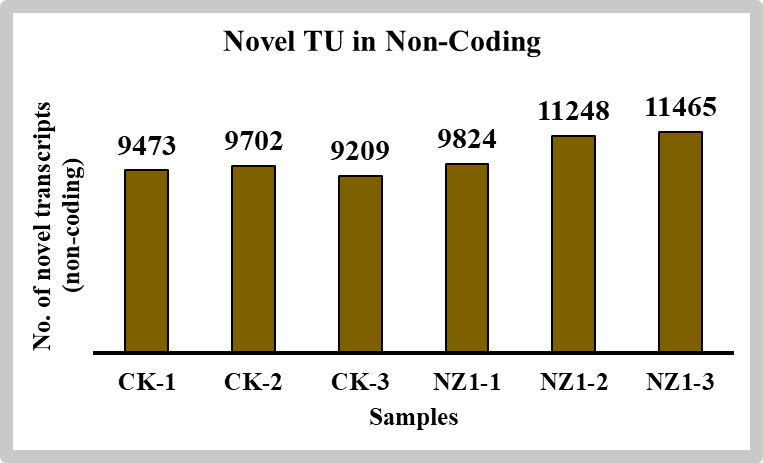

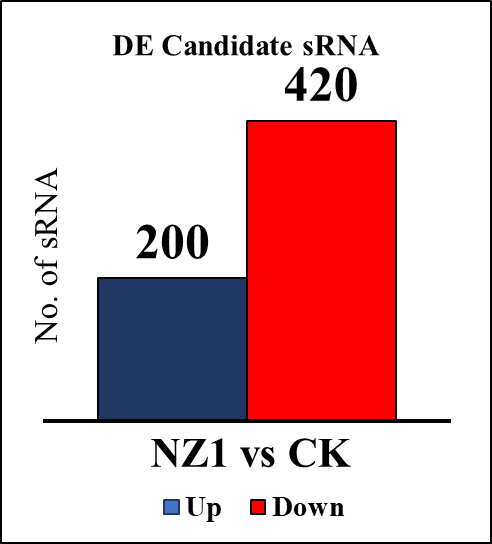

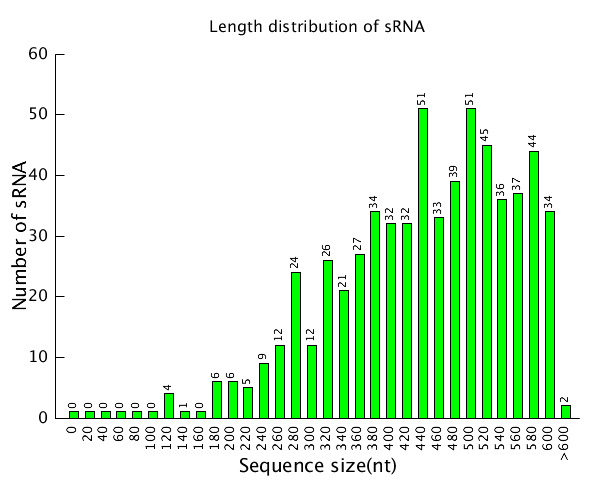


**Continued…**

**d**

**Fig. S19** a) Total number of novel transcripts discovered based on the transcriptome data analysis, which includes both novel transcripts in coding and non-coding regions; b) Total number of differentially expressed sRNA in control and treatment samples; c) distribution of the total number of sRNA’s and their length; d) Top differentially expressed sRNA’s represented by its candidate-ID along with its start and ending regions.
